# *PagGRF11* Overexpression Promotes Stem Development and Dwarfing in *Populus*

**DOI:** 10.3390/ijms23147858

**Published:** 2022-07-16

**Authors:** Yanting Tian, Ye Zhao, Yuhan Sun, Yousry A. El-Kassaby, Guoyong Song, Yueqi Mi, Juan Han, Yun Li

**Affiliations:** 1 Engineering Technology Research Center of Black Locust of National Forestry and Grassland Administration, National Engineering Research Center of Tree Breeding and Ecological Restoration, Key Laboratory of Genetics and Breeding in Forest Trees and Ornamental Plants of Ministry of Education, College of Biological Sciences and Technology, Beijing Forestry University, Beijing 100083, China; yttian@bjfu.edu.cn (Y.T.); zhaoye@bjfu.edu.cn (Y.Z.); sunyuhan@bjfu.edu.cn (Y.S.); myqbjfu@163.com (Y.M.); hanjuan@bjfu.edu.cn (J.H.); 2 Department of Forest and Conservation Sciences, Faculty of Forestry, The University of British Columbia, 2424 Main Mall, Vancouver, BC V6T 1Z4, Canada; y.el-kassaby@ubc.ca; 3 College of Material Science and Technology, Beijing Forestry University, Beijing 100083, China; songg@bjfu.edu.cn

**Keywords:** *PagGRF11*, *atgrf*, xylem differentiation, dwarfing, *Populus*, *CCCH39*

## Abstract

Poplar is not only an important woody plant, but also a model species for molecular plant studies. We identified *PagGRF11* (pAxG07Gg0005700), a homolog of the Arabidopsis *AtGRF1* (AT4G37740) and *AtGRF2* (AT2G22840) gene. We transformed the poplar clone “84K” with *PagGRF11*, and the transgenic overexpressed plants (*PagGRF11-OE*) showed plant height reduction (dwarfing), stem diameter increase, internode shortening, and larger leaf area. The *Arabidopsis* overexpression line *grf-oe* (Overexpression of *PagGRF11* in *Arabidopsis*), mutant line *atgrf* (*a loss-of-function mutant of the AtGRF1 gene of Arabidopsis thaliana*), and mutant trans-complementary line *atgrf+oe* (*overexpression of PagGRF11 in mutant plants* (*atgrf*)) also showed different leaf size phenotypes. Further, tissue sections revealed that increased xylem production was the main cause of stem thickening. Transcriptome differential expression analysis of *PagGRF11* overexpressed and control plants showed that *PagGRF11* promoted *CCCH39*(*C3H39*) expression. The expression profile of *CCCH39* in different tissues showed that it was highly expressed in xylem. Yeast single hybrid and instantaneous double luciferase assay results showed that *PagGRF11* directly transcribed and activated *CCCH39* expression through interaction with cis-acting element GARE (TCTGTTG), thus promoting xylem development. This is the first finding that GRF positively regulates xylem development through *CCCH39* expression activation and further suggests that *PagGRF11* is a potential target for increasing wood yield.

## 1. Introduction

Poplar is one of the fastest-growing woody species with the largest cultivation area and the highest wood production in the world, and is characterized by its fast growth and high yield [1,2]. Improvement of secondary growth and wood properties through modern biotechnology is a powerful way to produce fast-growing and high-wood quality species. Therefore, strengthening research on the molecular mechanism of forest tree growth and development, especially the molecular mechanism of secondary growth, can provide effective theoretical guidance and technical support for woody species cultivation.

Growth regulating factors (GRF) are a class of plant-specific transcription factors that play an important role in plant growth and development regulation [3,4]. Their proteins are highly conserved, especially the N-terminal region, which contains two specific structural domains; namely, Gln-Leu-Gln (QLQ, glutamine, leucine, glutamine, IPR014978) and Trp-Arg-Cys (WRC, tryptophan, arginine, cysteine, IPR014977) [5,6,7]. In rice, earlier study showed that GRF rapidly responded to GA treatment, suggesting that it might be associated with growth and development [8]. GRFs have also been found in several plant species such as *Arabidopsis thaliana*, *Zea mays*, *Medicago truncatula*, *Brassica rapa*, and *Populus trichocarpa*, representing a large gene family, with 9, 12, and 19 genes in *A. thaliana, O. sativa, and P. trichocarpa*, respectively [9,10,11].

GRFs influence on stem elongation has also been observed. In rice, *OsGRF1* is involved in mediating GA-dependent stem growth and in regulating heading time [8,12]. The accumulation of *GRF4* in rice resulted in a half-dwarf plant with increased yield, which was associated with the provision of N through increased N use efficiency [13,14]. In addition, GRF upstream regulator, miR396, and GRF-interacting protein GIF were also involved in stem elongation regulation [3,15,16,17]. miR396 target site mutations in *OsGRF4* slightly increased rice plant height, and further studies revealed that the reduced height of 35S:OsMIR396d rice was due to the endogenous cell growth being inhibited, causing the cells to become shorter and resulting in shorter stem internodes, while the number of internodes did not change. *OsGRF6* can promote GA synthesis and signal transduction, which in turn regulates rice plant height [12,16]. In maize, overexpression of Zm-rGRF1 resulted in dwarfism, probably due to strong ectopic expression of *Zm-rGRF1* interfering with the stem elongation process [18,19]. However, the molecular mechanisms of GRF development and formation in woody plants’ xylem are incomplete.

The zinc finger transcription factors are the largest family of transcription factors in plants and are key regulators of a variety of biological processes including morphogenesis, signal transduction, and environmental stress [20]. The *CCCH* (*C3H*) gene family is also a family of zinc finger proteins that regulate gene expression by directly binding to mRNA and contain a typical C3H-type motif, and members of this family have already been identified in several organisms from yeast to humans [21,22]. Compared to the largely well-characterized *CCCHs* in animals, only a small number of CCCH proteins have been functionally characterized in *Arabidopsis* and rice [23]. Members of higher plant-specific CCCH subfamily in *Arabidopsis thaliana, Populus*, and other plant species are known to play conserved roles in plant secondary growth, xylem development, and stress response [21,24]. *AtC3H14* and *AtC3H15* have recently been shown to act as the master regulators for secondary cell wall biosynthesis in Arabidopsis. Overexpression of *PuC3H35* was shown to increase drought tolerance through the promotion of ROS-scavenging and lignification of *Populus* roots [22]. However, there are few studies on the gene function of *CCCH39* and its upstream regulators are unclear.

Poplar is an important tree genus with rapid growth and high wood production. Here, the role of the GRF family gene PagGRF11 is characterized in “84K” poplars. Overexpression of PagGRF11 exhibited a thickened and shorter stem node, dwarfing, and larger leaf phenotype. Tissue sections were performed and revealed that widening of the xylem band was the main reason for the stem thickening. Meanwhile, stem compositional analysis showed that overexpression of PagGRF11 was able to increase the lignin content. At the same time, C3H39, which is highly expressed in xylem, can also be up-regulated by PagGRF11. The generated results are expected to provide important insights into the construction of regulatory networks related to woody plant development, especially wood formation, as well as new target sites for wood yield improvement.

## 2. Results

### 2.1. Identification of PagGRF11 in 84K

**Figure 1:**
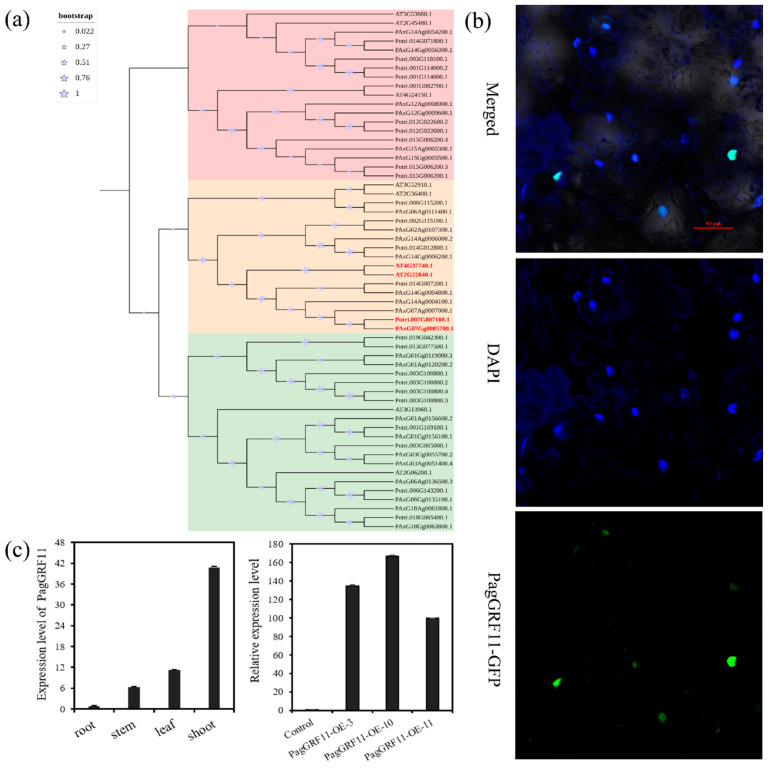
Evolutionary analysis, subcellular localization and expression analysis. (**a**) Neighbor-joining phylogenetic tree of PagGRF-related proteins from “84K”, *Arabidopsis thaliana,* and *Populus*. The tree was constructed from an alignment conducted using MEGA X with 1000 bootstrap replicates; the bootstrap value for each node is shown on the tree with star symbol. (**b**) *PagGRF11-GFP* fusion reporter in N. Subcellular localization of the tobacco cells by confocal laser microscopy. GFP, DAPI and merged images are shown (scale bar = 50 μm). GFP: Green fluorescent signal of *PagGRF11-GFP* protein, DAPI: Blue nucleus fluorescence signal. (**c**) Expression patterns of *PagGRF11* in different tissues of “84K” poplar (left). The horizontal axis represents different tissues, and the vertical axis represents qRT-PCR value. Expression levels of *PagGRF11* in control and transgenic plants (right). The vertical axis represents the values of qRT-PCR and the horizontal axis represents different samples. Control: “84K” populus carried the pBI121 empty vector. *PagGRF11-OE-3*, *PagGRF11-OE-10*, *PagGRF11-OE-11*: Overexpression of *PagGRF11* in “84K” poplar.

A total of 19 GRF genes were identified from the *Populus trichocarpa* genome (*Populus_trichocarpa*-Ensembl Genomes 53 http://plants.ensembl.org/Populus_trichocarpa/Info/Index accessed on 9 October 2020) and compared to clone “84K” genome (https://www.ncbi.nlm.nih.gov/bioproject/?term=Populus%20alba%20x%20Populus%20tremula%20var.%20glandulosa%20clone%2084K accessed on 22 October 2020) with a similarity expectation value of 1.0 × 10^−100^. We screened 24 GRF gene family members from the “84K” genome, among them, “84K” *PagGRF11* (PAxG07Gg0005700.1) is homologous to *Populus trichocarpa PtGRF11* (Potri.007G007100.1) and *Arabidopsis AtGRF1/2* (AT4G37740, AT2G22840), indicating a possible similar gene function (Figure 1a). *PagGRF11* encodes 419 amino acids containing a QLQ structural domain and a WRC structural domain specific to the *GRF* transcription factor gene family, implying that *PagGRF11* is a member of the *PagGRFs* gene family.

Furthermore, the *PagGRF11* subcellular localization showed that it has a strong green fluorescent signal in the nucleus of tobacco leaf subepidermal cells, indicating that it contains a nuclear localization signal (Figure 1b). We also examined the expression pattern of *PagGRF11* in different tissues through quantitative reverse transcription polymerase chain reaction (qRT-PCR) (Figure 1c) and the results showed that it was expressed at the highest level in shoots and the lowest level in roots, suggesting that its expression may contribute to stem growth and development.

### 2.2. Overexpression of PagGRF11 in “84K” Leads to Plant Dwarfism and Stem Thickness

**Figure 2:**
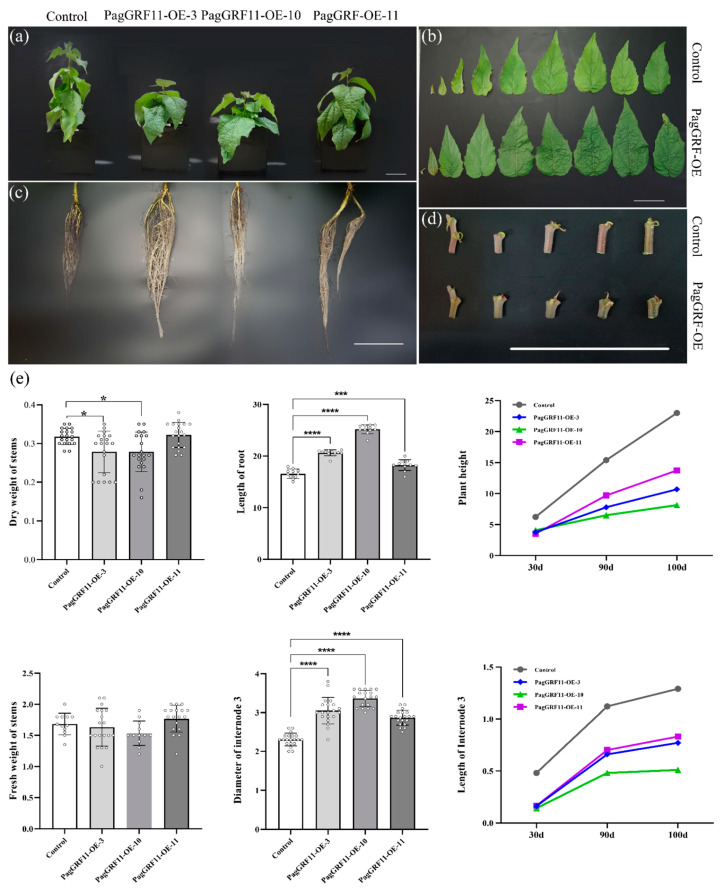
Morphological characterization of *Pag-GRF11*-overexpressing transgenic plants. (**a**–**d**) General characteristics of 3-month-old plants overexpressing *PagGRF11* and control. Control: “84K” populus carried the pBI121 empty vector (scale bar = 5 cm). (**a**) Plant height, (**b**) leaf, (**c**) root, and (**d**) stem internode of control and PagGRF11 transgenic plants. (**e**) Three-month-old plants were used to measure stem dry weight, stem fresh weight, plant height, third internode diameter, root length, stem third internode length. * denotes *p* < 0.0194, *** denotes *p* < 0.0002, **** denotes *p* < 0.0001.

To further investigate the role of *PagGRF11* in growth and development, *PagGRF11* overexpressed transgenic plants were constructed with a 35S promoter. We obtained 8 *PagGRF11-OE* (overexpression of *PagGRF11* in “84K” poplar) transgenic lines; among them, *PagGRF11-OE* lines 3, 10, and 11 expressed significantly higher ploidy than the control plants by 130-, 160-, and 90-fold, respectively, and were selected for further analyses (Figure 1c). The three transgenic lines (*PagGRF11-OE-3*, *PagGRF11-OE-10*, and *PagGRF11-OE-11*) had different morphological characteristics from the control and these differences were quantified statistically for plant height, stem third internode length and diameter, and plant dry and fresh weight (Figure 2a–e). The results indicated that the three transgenic lines were significantly shorter (i.e., dwarf) than the control. Similarly, after 100 d growth, the stem third internode lengths of the transgenic line were significantly shorter than the control (Figure 2d,e). Compared to the control, the stem diameter of the third internode of the transgenic lines showed a 32.61, 46.09, and 26.09% increase for *PagGRF11-OE-3*, *PagGRF11-OE-10*, and *PagGRF11-OE-11*, respectively (Figure 2e), while their leaves were longer and wider (Figure 2b), and their roots were longer (range: 10.072–24.487%). Two of the transgenic plants (*PagGRF11-OE-3* and *PagGRF11-OE-10*) produced reduced dry weight (range: 12.30–20.19%) and fresh weight (range: 3.03–5.88%) as compared to control, while the third (*PagGRF11-OE-11*) produced similar dry weight and fresh weight to the control (Figure 2e).

### 2.3. Effect of PagGRF11 Overexpression on Stem Xylem Development

**Figure 3:**
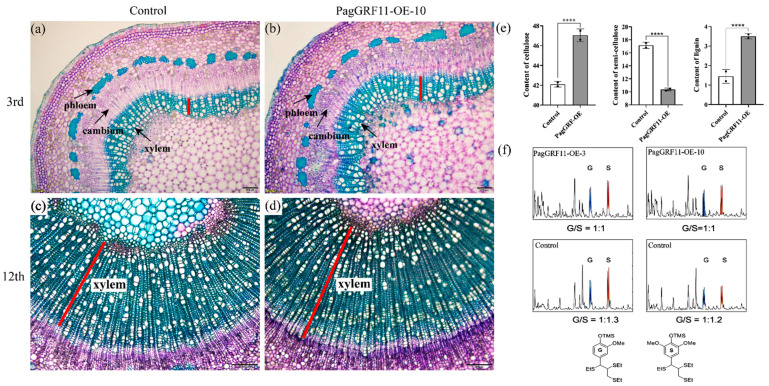
Cross-sections of stems at the 3rd and 12th internode of *PagGRF11* and control plants. (**a**,**b**) Cross section of stem 3rd internode of *PagGRF11* and control plants; (**c**,**d**) Stem 12th internode cross section of *PagGRF11* and control plants; (**e**) Analysis of the stem composition of transgenic and control plants; **** denotes *p* < 0.0001. (**f**) Thiolysis analysis of lignin monomers in 3-month-old *PagGRF11-OE* and control plants. G: G-type lignin monomer, S: S-type lignin monomer.

To investigate stem thickening in the transgenic plants, we sampled the stem 3rd and 12th internodes from 3-month-old plants (transgenic and control) and histologically stained their cross-section. The results showed that *PagGRF-OE* plants exhibited a significant increase in xylem thickness, suggesting that *PagGRF11* may be involved in the early lignin synthesis pathway (Figure 3a–d). Analysis of the stem composition of the transgenic and control plants showed that the transgenic plants’ lignin and cellulose content were significantly higher and the hemi-lignin content was significantly lower than that of the control.

To further compare whether the proportion of lignin monomer types differed between the control and *PagGRF11-OE* plants, we examined the proportion of G- and S-type lignin monomers by Thiolysis (Figure 3f) and a slight decrease in the S-type lignin monomers proportion was found in *PagGRF11-OE* plants, further confirming *PagGRF11* involvement in the lignin regulatory pathway.

### 2.4. Overexpression of PagGRF11 in Arabidopsis Causes Leaf Enlargement

**Figure 4:**
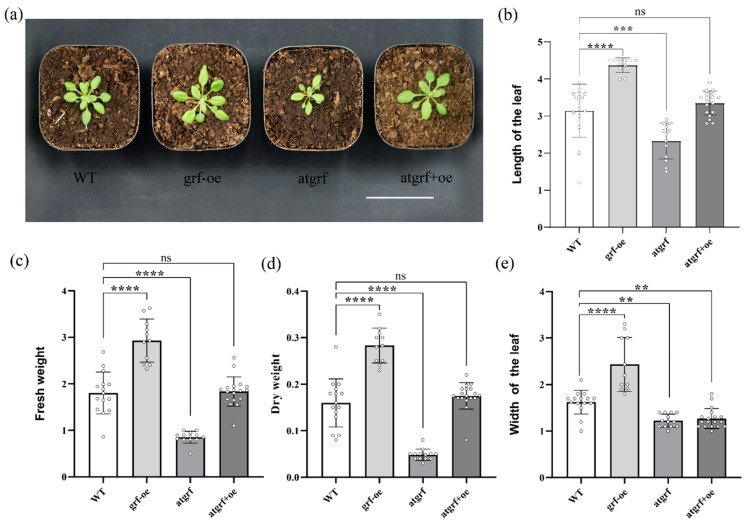
Phenotype of *PagGRF11* transformed Arabidopsis plants. (**a**) Morphological characterization of *WT*, *grf-oe*, *atgrf*, *atgrf+oe*. WT: Wild-type *Arabidopsis Columbia 0*. *grf-oe*: Overexpression of *PagGRF11* in *Arabidopsis*. *atgrf*: *A loss-of-function mutant of the AtGRF1* gene of *Arabidopsis thaliana*. *atgrf+oe*: The *PagGRF11* gene was overexpressed in mutant plants (*atgrf*). (**b**–**e**): Statistics of leaf length (**b**), leaf width (**e**), dry weight (**d**) and fresh weight (**c**) of *WT*, *grf-oe*, *atgrf*, *atgrf+oe* 4 transgenic lines. ** denotes *p* < 0.0077, *** denotes *p* < 0.0002, **** denotes *p* < 0.0001, “ns” denotes the difference is not significant.

To investigate whether *PagGRF11* overexpression in other plants has a similar function to that in poplar, we overexpressed *PagGRF11* in *Arabidopsis* to obtain a transgenic line *grf-oe*. The results indicated that the *grf-oe* exhibited a similar phenotype to that of transformed poplar with enlarged leaves compared to the *Arabidopsis* wild type (WT) (Figure 4a). Compared to the WT, the original *atgrf* mutant (a loss-of-function mutant of the *AtGRF1* gene of *Arabidopsis*) showed smaller leaves. However, *atgrf+oe* lines were obtained after *PagGRF11* was overexpressed in *atgrf* plants, and the transformed *atgrf+oe* produced larger leaves than those of *atgrf* (Figure 4b–e). For *Arabidopsis thaliana* plants grown for two weeks, the dry and fresh weights of *grf-oe* were significantly higher than the WT, while the dry weight was significantly lower than the WT (Figure 4c,d). These results suggest that *PagGRF11* is capable of producing a leaf phenotype similar to that of *Arabidopsis AtGRF1*, implying that the homologous pair of *PagGRF11* and *AtGRF1* are functionally similar. On the other hand, a significant expansion of the GRF gene family from lower to higher plants has been documented in previous studies [5,25]. Here, we found that *PagGRF11* did not result in phenotypic changes other than those in *Arabidopsis* leaves, whereas *PagGRF11* transgenic poplars had more phenotypic changes, such as a wider xylem band, suggesting that *PagGRF11* has a secondary growth regulatory function unique to woody plants. Further studies are needed to determine this regulatory mechanism.

### 2.5. PagGRF11 Interacted with CCCH39 Promoter Sequences and Induced Their Expression

**Figure 5:**
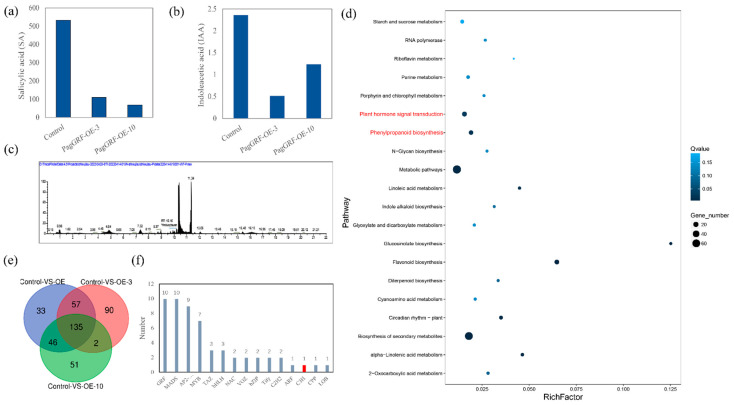
Transcriptome analysis of PagGRF11 overexpressing plants and control. (**a**–**c**) Contents of salicylic acid and indoleacetic acid in control, PagGRF-OE-3, and PagGRF-OE-10 were detected by high performance liquid chromatography. (**d**) KEGG enrichment analysis of 135 DEGs. The size of the dots represents the number of genes (the larger the dot, the more genes are enriched). The color of the dots indicates the enrichment significant level (the darker the color, the smaller the Q value, and the more significant the enrichment degree is). (**e**) Venn diagram analysis of three comparison groups: Control-VS-OE, Control-VS-OE-3, and Control-VS-OE-10.OE: *PagGRF11-OE-3* and *PagGRF11-OE-10* mixed samples; OE-3: *PagGRF11-OE-3* lines; OE-10: *PagGRF11-OE-10* lines. (**f**) All transcription factor families and numbers in the 135 differential genes; the vertical axis represents the number of transcription factors, and the horizontal axis represents the transcription factor families.

GRFs, as a class of transcription factors unique to plants, are involved in a variety of developmental processes by regulating the expression of downstream genes. To find out which genes are regulated by *PagGRF11*, we selected one-month-old control (control) and transgenic plants (*PagGRF11-OE-3* and *PagGRF11-OE-10*) for comparative transcriptome analysis. There were three comparison groups: 1) mixed samples of lines *PagGRF11-OE-3* and *PagGRF11-OE-10* compared with controls (Control-vs-OE), 2) the transgenic line *PagGRF11-OE-3* compared with control (Control-vs-OE-3), and 3) the transgenic line *PagGRF11-OE-10* compared with control (Control-vs-OE-10) and were screened for 234, 284, and 271 significantly differentially expressed genes (DEG), respectively (Figure 5e and Table S2). Among these DEGs, 135 co-occurred in the three combinations. KEGG enrichment analysis revealed that the top 20 pathways were significantly enriched in the lignin biosynthesis pathway, results consistent with our previous statistical analysis of this trait (Figure 5d). Further analysis revealed that 56 transcription factors were significantly enriched into 15 transcription factor families. Of these, the CCCH gene family was associated with lignin biosynthesis. In our transcriptome results, only *CCCH39* was found to be significantly enriched (Figure 5f). KEGG enrichment analysis was also annotated to the hormone signaling pathway. We examined the endogenous hormone content of control and *PagGRF11-OE* plants using high performance liquid chromatography and found that *PagGRF-OE-3* and *PagGRF-OE-10* plants had significantly lower IAA and salicylic acid contents than the control (Figure 5a–c). Meanwhile, KEGG enrichment analysis was annotated to the phenylpropanoid biosynthesis pathway, which may correlate with phenotypic changes in the wider xylem band (Figure 5d).

In previous studies, the GRF family of proteins had a single C-X8- 9-C-X10-C-X2-H-type zinc-binding motif in the WRC domain corresponding to the Hordeum suppressor (HRT) motif of the barley transcriptional repressor protein, which is thought to bind to the GA response element (GARE) motif [12,26,27]. Analysis of the *CCCH39* promoter revealed that it contains a TCTGTTG element; therefore, we speculate that *PagGRF11* regulates *CCCH39* gene expression by binding to the GARE (TCTGTTG) motif of the *CCCH39* promoter.

### 2.6. PagGRF11 Transcriptional Activation Regulates CCCH39 Expression

**Figure 6:**
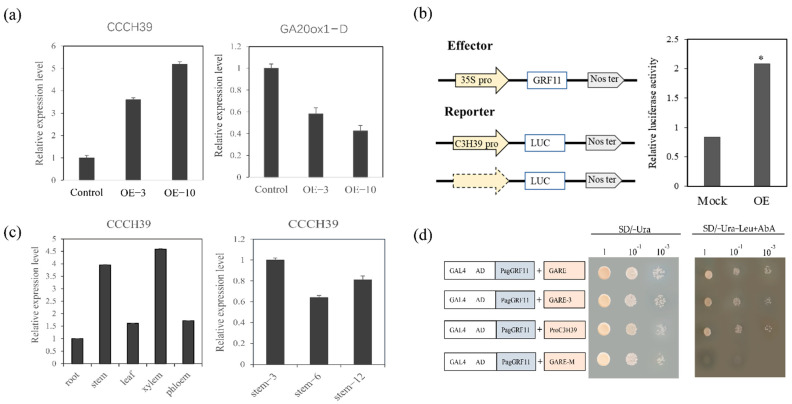
Overexpression of *PagGRF11* up-regulated *CCCH39* expression and resulted in plant dwarfing and increased lignin. (**a**) Expression level analysis of *CCCH39* and *GA20ox1-D* in *PagGRF-OE* and control. OE-3: *PagGRF11-OE-3*, OE-10: *PagGRF11-OE-10*. (**b**) Dual-luciferase assay showing the increase of *CCCH39* by the *PagGRF11* effector construct compared to the control effector construct. Effect: Effect vectors; Reporter:Report vector. Mock: Effectors co-transform tobacco with empty reporters; OE: Effectors co-transform tobacco with C3H39 pro reporters. * denotes *p* = 0.0001. (**c**) Analysis of expression patterns of CCCH39 in different tissues. (**d**) Yeast single hybrid confirmed that *PagGRF11* could directly activate the expression of *CCCH39*. Four yeast reporters were constructed: GARE (TCTGTTG), GARE-3 (TCTGTTCTCTGTTGTCTGTTG), ProC3H39 (*CCCH39* promoter sequence length 533 bp), GARE-M (TCCCCCC), and the PagGRF11-AD fusion expression vector was co-transfected into yeast respectively. Growth on SD/-Ura and SD/-Ura-Leu+AbA medium.

Luciferase reporters with *CCCH39* promoters cloned from the hybrid poplar “84K” were separately co-transformed into *Nicotiana tabacum* with the *PagGRF11* effector; the *CCCH39* reporter produced higher than 2-fold luciferase activity compared to the mock control, which was consistent with the changes in its expression observed by RNA-seq and qRT-PCR (Figure 6a,b).

In a previous study, GARE (TCTGTTG) was determined to be the binding element for *OsGRF7* [28]. Thus, we next verified PagGRF11 binding to the target gene *CCCH39* in a yeast single-hybrid assay. All four-yeast reporter PagGRF11-AD fusion expression vectors of GARE (TCTGTTG), GARE-3 (TCTGTTCTCTCTGTTGTCTGTTG), ProC3H39 (*CCCH39* promoter sequence length 533 bp), and GARE-M (TCCCCCC) were constructed by the GAL4 system and transformed yeast together, and all were found to grow normally except for the TCTGTTG mutation site (Figure 6d), suggesting that *PagGRF11* is able to bind directly to the GARE element on the *CCCH39* promoter.

*CCCH39* expression analysis at different sites showed that *CCCH39* was most highly expressed in the xylem. The expression level of *CCCH39* gene was also analyzed in the stem 3rd, 6th and 12th internodes of two-month-old wild-type seedlings. The 3rd internode of the stem includes primary growth vascular tissue and is younger. The stem 12th internodes have well developed secondary phloem tissues and secondary xylem vessels, as well as fibers with well lignified secondary walls. The analysis showed that *CCCH39* expression was highest in the stem 3rd internode, implying that *CCCH39* is highly expressed in the young stem (Figure 6c). This is consistent with previous findings on the CCCH gene family [23], suggesting that *CCCH39* has a positive regulatory effect on xylem synthesis. Additionally, previous studies have suggested that the gibberellin synthesis gene *GA20ox* has a positive regulatory effect on plant height. Our results showed that GA20ox-D expression was down-regulated by RNA-seq and qRT-PCR in *PagGRF11-OE* plants compared to control, implying that *PagGRF11* may regulate poplar plant height by participating in the gibberellin synthesis pathway (Figure 6a).

## 3. Discussion

The GRF gene family, a plant-specific class of transcription factor families associated with growth and development, has an important role in plant growth and developmental processes. There are 19 members of the GRF gene family in *Populus trichocarpa*, which is 2-fold higher than the number of *AtGRFs* genes in Arabidopsis (9). In the “84K” poplar genome, 24 members of the GRF gene family were found, which is 2.67-fold more than the number of members of the Arabidopsis GRF family, and even more than the genome of the *Populus trichocarpa*. This may be related to the fact that the “84K” poplar has two different sets of chromosomes. However, in any case, these ratios are much greater than the 1.4–1.6 ratio between poplar homologs and Arabidopsis genes in comparative genomics [29], suggesting that they have greater amplification in woody compared to herbaceous plants. In our study, *PagGRF11* was found to have similar gene functions in *Arabidopsis* and *Populus* in regulating leaf size. However, *PagGRF11* poplar transgenic plants had wider xylem bands and dwarfed plants, suggesting that *PagGRF11* is involved in secondary growth of woody plants, which is a woody plant-specific growth and development process that distinguishes herbaceous plants. Suggesting that GRF has a specific biological function in the evolution from lower to higher plants.

The structure of GRFs is very conserved, but they have their own characteristics in terms of motifs. For example, previous studies have shown that *PagGRF12a* negatively regulated lignin synthesis [5,25]. In contrast, in our study, *PagGRF11* positively regulated lignin synthesis, probably because although it is the same gene family, different members are involved in different signaling pathways.

Despite the important role of GRFs in secondary plant growth and development, it is unclear how they affect lignin formation. Here, our analysis of the expression pattern of *CCCH39* (downstream genes of *PagGRF11*) in different parts of “84K” poplar revealed that *CCCH39* was highly expressed mainly in the xylem, suggesting that it affects lignin accumulation. In contrast, *PagGRF11* could affect lignin synthesis and accumulation through *CCCH39* regulation. Further studies revealed that the proportion of S-type monomers tended to decrease and the total amount of lignin increased in *PagGRF-OE* plants, suggesting that *PagGRF11* may affect the production of S-type monomers or their polymerisation through *CCCH*, but the exact process in which it affects monomer synthesis is unclear and needs further study. Lignin, as an important biomolecule of great economic and application value and its anabolic pathway has also received great attention, thus it is important to investigate the molecular mechanism of its synthesis.

Plant CCCH zinc finger proteins regulate plant growth and development mainly at the post-transcriptional level and are considered to be an important gene family associated with wood development [20,30]. The family underwent a large number of replication events in the poplar genome and therefore its family members (91) are significantly higher than those of herbaceous plants such as *Arabidopsis thaliana* (68) and rice (67) [20,23]. However, the specific functions of some of its members are unclear. In previous studies, *PuC3H35* was found to be involved in mediating proanthocyanidin (PA) biosynthesis and lignin in poplar roots in response to drought stress in *Populus ussuriensis*, and overexpression of *PuC3H35* promoted PA and lignin biosynthesis and vascular tissue development, resulting in enhanced tolerance to drought stress by means of antioxidant and mechanical support [22]. Overexpression of *PdC3H17* in poplar caused dwarfism, produced more xylem vascular cells, resulted in higher stem water potential and showed increased photosynthesis [31]. Analysis of *CCCH39* expression pattern in different tissue parts showed that it was highly expressed in the xylem. Analysis of *CCCH39* expression in different stem developmental stages also showed that it was highly expressed in relatively young stems with well lignified secondary walls. These results are consistent with previous studies and suggest that it is most likely to be involved in the lignin synthesis pathway. In order to reveal more specific biological functions of *CCCH39*, we are undertaking a functional study of the “84K” poplar transgene, with a view to further elucidating how the *PagGRF11-CCCH39* module regulates xylem formation and affects lignin accumulation in specific ways.

## 4. Materials and Methods

### 4.1. Vector Construction and Plant Transformation

*PagGRF11* homologs’ amino acid sequences were retrieved from the National Center for Biotechnology Information (NCBI) database. *PagGRF11* full coding and promoter sequences were cloned in the hybrid poplar “84K” using the primer clones listed in Table S1. *35S::PagGRF11* constructs were generated using PBI121 vectors. PCR amplification was performed using the primers listed in Table S1. *PagGRF11* stop codon was replaced with the nucleotide sequence encoding the GFP domain (encoding a fluorescent protein), and the *PagGRF11-GFP* amplicon was then inserted into the PBI121 vector to generate the *35S::PagGRF11-GFP* construct. *35S::PagGRF11* constructs were introduced into *Populus alba×P.glandulosa* via the leaf disc method [32]. *35S::PagGRF11* constructs were transformed by infesting *Arabidopsis* inflorescences with *Arabidopsis Columbia 0* and *atgrf* mutants (*AtGRF1* loss-of-function mutants). All transgenic plants were identified and verified by PCR amplification and expression. *Atgrf* mutants were obtained from the Chinese Arabidopsis Mutant Sharing Center (Arashare, Fuzhou, China). All transgenic *Populus* and *Arabidopsis* lines were kept in the Tissue Culture Room of the High Precision Innovation Centre at Beijing Forestry University, China. All plants were grown at 24 °C with 16 h of light and 8 h of darkness.

### 4.2. RNA Extraction and RT-qPCR Analysis

The cetyltrimethylammonium bromide (CTAB, Coolaber, Beijing, China) method was used to extract total RNA [33]. cDNA reverse transcription was carried out using the reverse transcription kit RR0316A from Takara Bio (Takara Dalian, China), according to the manufacturer’s instructions. RT-qPCR was carried out by a one-step method using the SYBR kit (AG, Hunan, China) according to the manufacturer’s instructions.

### 4.3. Histochemical and Histological Analyses

Cross sections of the basal stems were produced from 3-month-old transgenic poplars and control. Briefly, 0.5 cm stem segments were submerged in ddH_2_O, then fixed in 7% agarose. Thin slices were cut to 35 μm thickness using a vibrating microtome (Leica EM UC7), stained with toluidine blue, and photographed under a microscope (Axio Imager A1, Carl Zeiss, Beijing, China).

### 4.4. Subcellular Localization Analysis

The *35S::PagGRF11::GFP* vector was constructed and transformed into *Agrobacterium tumefaciens* GV3101. Young (4-week-old) tobacco plant leaves were used for needleless syringe infiltration following [5]. After injection and incubation in the dark for 24 h, plants were then transferred to light for 2 days, followed by observing and photographing with a laser confocal microscope (Nikon Super Resolution Laser Scanning Confocal Microscope (Instrument No. A14000021, Tsinghua University, Beijing, China).

### 4.5. Development and Growth Analysis

Three-month-old control and transgenic plants were used as the experimental material for morphological analysis to determine plant height, stem diameter, and root length. Fresh biomass and dry weight yield of control and transgenic positive plants were examined after 100 d of growth in hydroponic culture in a light incubator. All stem dry tissues were ground and the lignin–lignin monomer ratio was quantified by the mercaptolysis. All stem dry tissues were ground and the lignin content was quantified by the Klason method and the supernatant was used to determine cellulase and hemicellulose content.

### 4.6. Transcriptome Analysis

Total RNA was isolated from shoot-tip tissue collected from three independent CK and *PagGRF11 OE-3* and *OE-10* plants (2-month-old) using the RNeasy Plant Mini Kit (Qiagen, Shanghai, China), followed by determining total RNA quantity and purity using Qubit RNA HS, and RNA integrity quality control using the 2100 RNA Nano/Pico kit. RNA sequencing libraries were constructed from transgenic and control plants (three biological replicates per sample). Raw data obtained from sequencing were filtered and the filtered clean reads were compared to the *Populus* genome (http://plants.ensembl.org/Populus_trichocarpa/Search/Results?species=Populus_trichocarpa;idx=;q=;site=ensemblunit accessed on 17 August 2021). Differential expression analysis was performed based on gene expression between samples, and GO and pathway functional analysis was performed on the screened differentially expressed genes.

### 4.7. Yeast One-Hybrid Assays

*PagGRF11* cDNA full-length was fused to the PAGDT7 (AD) vector (*PagGRF11-AD*). Yeast reporters were constructed by fusing promoter regions or transcriptional activation element sequences to the pAbAi vector. Four yeast reporters (*CCCH39* promoter 500 bp, TCTGTTG motif element, TCTGTTGTCTGTTGTCTGTTG motif element, and TCCCCCC motif element) were constructed to transform the yeast strain Y1HGold to generate a Bait-Reporter yeast strain. PagGRF11-AD was transformed into the Bait-Reporter yeast strain from the previous step and all strains were grown on SD selection medium (SD/-His-Leu) for 7 days. Y1H assay was performed according to the manufacturer’s instructions (Code No. 630,491 Takara, Dalian, China).

### 4.8. Dual Luciferase Reporter Assay of Transient Expression

The dual luciferase reporter transcriptional activation assay was performed as previously reported [22]. Briefly, the CDs region of *PagGRF11* and the *CCCH39* promoter region (2000 bp upstream of ATG) were separately cloned into effectors (pGreen-62-sk, 35S::transcription factor) and reporters (pGreen-0800-LUC, promoter-luciferase) in a vector. The effector and reporter were then co-transformed into tobacco leaves and after 2 d incubation, the dual luciferase activity was measured using a dual luciferase reporter system (Promega, GloMax 20/20 Luminometer Beijing, China).

## 5. Conclusions

We transformed the poplar clone “84K” with *PagGRF11*, and the transgenic overexpressed plants showed a reduction in plant height (dwarfing), an increase in stem diameter, internode shortening, and a larger leaf area. Further, tissue sections revealed that increased xylem was the main cause of stem thickening. The expression profile of *CCCH39* in different tissues showed that *CCCH39* was highly expressed in xylem. Yeast single hybrid and instantaneous double luciferase assay results showed that PagGRF11 directly transcribed and activated *CCCH39* expression through interaction with cis-acting element GARE (TCTGTTG), thus promoting xylem development. This is the first finding that GRF positively regulates xylem development through *CCCH39* expression activation and further suggests that PagGRF11 is a potential target for increasing wood yield.

## Data Availability

RNAseq data is being uploaded to https://ngdc.cncb.ac.cn/omix/database accessed on 15 July 2022. All data are included in the article or in supplementary data published online. Data supporting the findings of this study are available from the corresponding author, Yanting Tian, upon request.

## Supplementary Materials

The following supporting information can be downloaded at: https://www.mdpi.com/article/10.3390/ijms23147858/s1.
