# Supplementary material for: PagGRF11 Overexpression Promotes Stem Development and Dwarfing in Populus
Source: Int J Mol Sci. 2022 Jul 16;23(14):7858. doi: 10.3390/ijms23147858 (PMC9323871; doi:10.3390/ijms23147858)
Supplement: Supplementary file 1 [file ijms-23-07858-s001.zip › ijms-1801723-supplementary.pdf]

Table S1. Primer sequences for the amplification of PagGRF11 and CCCH39

Primer	Sequence
PagGRF11-F	5' ATGGACTTTGGGGTTCTTGG 3'
PagGRF11-R	5' CTAACCATTTTCTTGATTACAGGG 3'
PagGRF11-GFP-F	5' ATGGACTTTGGGGTTCTTGG 3'
PagGRF11-GFP-R	5' ACCATTTTCTTGATTACAGGGAT 3'
TUB-F	5' CGGTGGTTCCATTCTTGC 3'
TUB-R	5' GGACCTGCCTCATCATACTC 3'
PagGRF11 qRT-PCR-F	5' TGGTGCCAATGTCTAACTCC 3'
PagGRF11 qRT-PCR-R	5' GCACGGAGGGTTTCTATGA 3'
PagC3H39 qRT-PCR-F	5' TGGAAAGCACTCAAGAAAACA 3'
PagC3H39 qRT-PCR-R	5' CTTCGACAACCACAACAAAGA 3'
PagC3H39 Promoter -F	5′TCTGTTG TCTGTTG TCTGTTG 3′
PagC3H39 Promoter -R	5′CAACAGACAACAGACAACAGA 3′
3-C3H39-F	5' AGCTTTCTGTTG TCTGTTG TCTGTTG C 3'
3-C3H39-R	5' TCGAGCAACAGACAACAGACAACAGA A 3'
1-C3H39-F	5' AGCTTTCTGTTG C 3'
1-C3H39-R	5' TCGAGCAACAGA A 3'
M-C3H39-F	5' AGCTTTCCCCCC C 3'
M-C3H39-R	5' TCGAGGGGGGGA A 3'

Table S2. Differentially expressed genes inPagGRF11-O× 10 compared with wild type

GeneID	Length	WT-× 10xpression	O× 10-× 10xpression	log2Fold Change(O× 10/WT)	Pvalue	Up/Down-Regulation	Blast nr
POPTR_008G220700v3	2022	261.38	685.76	1.1543	1.83×10^{-16}	Up	gi|566185340|ref|XP_006380149.1|/3.4e-89/hypothetical protein POPTR_0008s22370g [Populus trichocarpa]
POPTR_011G048100v3	1226	6.63	39.49	1.48087	1.37×10^{-9}	Up	gi|566193733|ref|XP_006377315.1|/1.8e-197/hypothetical protein POPTR_0011s04790g [Populus trichocarpa]
POPTR_003G189100v3	1465	118.3	31.37	-1.2095	1.52×10^{-6}	Down	gi|566163438|ref|XP_006385961.1|/1.2e-193/hypothetical protein POPTR_0003s18820g [Populus trichocarpa]
POPTR_006G255900v3	1763	153.45	69.89	-1.1083	3.11×10^{-11}	Down	gi|224089276|ref|XP_002308672.1|/6.1e-105/adenine phosphoribosyltransferase 2 family protein [Populus trichocarpa]
POPTR_009G065000v3	1973	170.1	449.23	1.03691	1.77×10^{-7}	Up	gi|224104019|ref|XP_002313284.1|/1.1e-302/hypothetical protein POPTR_0009s06940g [Populus trichocarpa]
POPTR_011G070000v3	900	23.63	162.65	1.64043	7.09×10^{-12}	Up	gi|566194230|ref|XP_002317398.2|/2.7e-74/hypothetical protein POPTR_0011s07000g [Populus trichocarpa]
POPTR_014G027300v3	989	6.47	78.26	2.37936	7.93×10^{-27}	Up	gi|566202085|ref|XP_006374919.1|/1.6e-143/basic helix-loop-helix family protein [Populus trichocarpa]
POPTR_013G154700v3	1381	2184.99	1103.31	-1.0306	2.39×10^{-10}	Down	gi|224124736|ref|XP_002319409.1|/1.4e-121/expansin S1 precursor family protein [Populus trichocarpa]//gi|224124736|ref|XP_002319409.1|/2.6e-148/expansin S1 precursor family protein [Populus trichocarpa]
POPTR_006G258400v3	980	12.75	72.89	1.22155	1.27×10^{-6}	Up	gi|566178429|ref|XP_002308682.2|/3.7e-148/hypothetical protein POPTR_0006s27460g [Populus trichocarpa]
POPTR_004G173400v3	1754	651.53	188.69	-1.4136	2.33×10^{-9}	Down	gi|224080606|ref|XP_002306177.1|/1.1e-226/nodulin family protein [Populus trichocarpa]//gi|224080606|ref|XP_002306177.1|/1.8e-202/nodulin family protein [Populus trichocarpa]//gi|224080606|ref|XP_002306177.1|/8.8e-293/nodulin family protein [Populus trichocarpa]//gi|743916026|ref|XP_011001982.1|/2.6e-156/PR× 10DICT× 10D: probable transporter mch1 [Populus euphratica]
POPTR_016G087000v3	853	75.98	33.8	-1.1062	2.22×10^{-8}	Down	gi|224138630|ref|XP_002322862.1|/2.0e-42/hypothetical protein POPTR_0016s08840g [Populus trichocarpa]
POPTR_015G087900v3	1523	17.85	91.97	1.63177	1.06×10^{-13}	Up	gi|224133768|ref|XP_002321656.1|/1.7e-245/hypothetical protein POPTR_0015s09940g [Populus trichocarpa]
POPTR_016G078000v3	1564	28.3	7.15	-1.2653	4.47×10^{-7}	Down	gi|566209265|ref|XP_002322819.2|/2.9e-273/hypothetical protein POPTR_0016s07860g [Populus trichocarpa]
POPTR_012G019700v3	2693	226.08	630.94	1.00803	5.58×10^{-6}	Up	gi|566196444|ref|XP_006376658.1|/5.1e-220/hypothetical protein POPTR_0012s02660g [Populus trichocarpa]//gi|566196446|ref|XP_006376659.1|/0.0e+00/hypothetical protein POPTR_0012s02660g [Populus trichocarpa]//gi|566196448|ref|XP_002317799.2|/0.0e+00/hypothetical protein POPTR_0012s02660g [Populus trichocarpa]
POPTR_001G075000v3	4399	34.16	89.79	1.03719	1.36×10^{-8}	Up	gi|224057908|ref|XP_002299384.1|/4.0e-245/leucine-rich repeat transmembrane protein kinase [Populus trichocarpa]
POPTR_011G049100v3	1103	26.86	189.97	2.05194	5.92×10^{-23}	Up	gi|566193736|ref|XP_006377316.1|/9.5e-186/hypothetical protein POPTR_0011s04810g [Populus trichocarpa]
POPTR_008G150300v3	1027	29.42	147.38	1.4358	1.66×10^{-9}	Up	gi|566184053|ref|XP_002312520.2|/8.0e-91/hypothetical protein POPTR_0008s14970g [Populus trichocarpa]
POPTR_001G106700v3	3688	161.67	563.42	1.49551	5.36×10^{-21}	Up	gi|224058559|ref|XP_002299544.1|/0.0e+00/hypothetical protein POPTR_0001s09120g [Populus trichocarpa]//gi|743852337|ref|XP_011029232.1|/2.6e-291/PR× 10DICT× 10D: putative leucine-rich repeat-containing protein DDB_G0290503 isoform X1 [Populus euphratica]
POPTR_008G110000v3	1337	157.78	414.04	1.027	2.79×10^{-8}	Up	gi|566183366|ref|XP_002312353.2|/1.9e-208/hypothetical protein POPTR_0008s10940g [Populus trichocarpa]
POPTR_003G213700v3	1351	60.28	11.03	-1.0375	3.66×10^{-5}	Down	gi|224073204|ref|XP_002304022.1|/3.9e-209/hypothetical protein POPTR_0003s21730g [Populus trichocarpa]
POPTR_016G140200v3	3263	71.02	303.22	1.15562	3.22×10^{-6}	Up	gi|743915385|ref|XP_011001641.1|/0.0e+00/PR× 10DICT× 10D: probable LRR receptor-like serine/threonine-protein kinase At1g06840 isoform X3 [Populus euphratica]//gi|743915385|ref|XP_011001641.1|/2.0e-267/PR× 10DICT× 10D: probable LRR receptor-like serine/threonine-protein kinase At1g06840 isoform X3 [Populus euphratica]//gi|743915385|ref|XP_011001641.1|/2.2e-242/PR× 10DICT× 10D: probable LRR receptor-like serine/threonine-protein kinase At1g06840 isoform X3 [Populus euphratica]
POPTR_001G114000v3	1094	101.6	395.45	1.30973	9.23×10^{-9}	Up	gi|566147919|ref|XP_002298083.2|/1.0e-98/hypothetical protein POPTR_0001s08310g [Populus trichocarpa]//gi|566147919|ref|XP_002298083.2|/2.1e-61/hypothetical protein POPTR_0001s08310g [Populus trichocarpa]//gi|566147919|ref|XP_002298083.2|/2.2e-113/hypothetical protein POPTR_0001s08310g [Populus trichocarpa]
POPTR_006G217900v3	1614	3.73	44.33	1.92478	4.62×10^{-15}	Up	gi|566177765|ref|XP_002308507.2|/1.0e-176/hydroxyproline-rich glycoprotein [Populus trichocarpa]//gi|743812549|ref|XP_011019305.1|/1.1e-188/PR× 10DICT× 10D: dual specificity protein kinase splA [Populus euphratica]
POPTR_003G176700v3	1592	223.06	42.5	-1.0822	1.79×10^{-5}	Down	gi|566163207|ref|XP_002303819.2|/1.9e-225/naregenin-chalcone synthase family protein [Populus trichocarpa]
POPTR_006G050800v3	946	82.65	22.02	-1.2194	1.26×10^{-6}	Down	gi|224090143|ref|XP_002308944.1|/6.4e-22/hypothetical protein POPTR_0006s04930g [Populus trichocarpa]
POPTR_003G150700v3	1434	127.39	62.35	-1.0594	3.63×10^{-12}	Down	gi|566162737|ref|XP_002304592.2|/4.6e-107/hypothetical protein POPTR_0003s15030g [Populus trichocarpa]

POPTR_006G054700v3	2662	71.6	16.02	-1.3995	2.12×10^{-8}	Down	gi|566174603|ref|XP_002308966.2|/0.0e+00/hypothetical protein POPTR_0006s05340g [Populus trichocarpa]
POPTR_011G062000v3	1353	103.6	45.11	-1.1483	1.44×10^{-9}	Down	gi|566194015|ref|XP_006377451.1|/6.3e-215/GDSL-motif lipase/hydrolase family protein [Populus trichocarpa]
POPTR_008G139100v3	1219	1065.62	4690.38	1.59948	5.81×10^{-15}	Up	gi|743785251|ref|XP_011024670.1|/7.1e-200/PR× 10DICT× 10D: purple acid phosphatase 4-like [Populus euphratica]
POPTR_004G075200v3	1847	129.05	56.8	-1.0861	9.70×10^{-9}	Down	gi|224079722|ref|XP_002305927.1|/1.6e-235/glutamate decarboxylase 1 family protein [Populus trichocarpa]//gi|224079722|ref|XP_002305927.1|/2.9e-292/glutamate decarboxylase 1 family protein [Populus trichocarpa]
POPTR_016G057600v3	1420	117.79	25.11	-1.1256	8.91×10^{-6}	Down	gi|566208913|ref|XP_002323325.2|/2.5e-197/hypothetical protein POPTR_0016s05800g [Populus trichocarpa]
POPTR_005G224200v3	1093	13.86	43.69	1.13415	1.85×10^{-7}	Up	gi|224085629|ref|XP_002307642.1|/4.0e-101/hypothetical protein POPTR_0005s24560g [Populus trichocarpa]
POPTR_004G054600v3	1581	202.15	12.17	-2.2254	2.21×10^{-18}	Down	gi|566165064|ref|XP_002305084.2|/2.1e-232/hypothetical protein POPTR_0004s05380g [Populus trichocarpa]
POPTR_003G001800v3	1476	29.9	126.36	1.03998	3.81×10^{-5}	Up	gi|743908784|ref|XP_011047854.1|/1.5e-265/PR× 10DICT× 10D: MLO-like protein 3 [Populus euphratica]
POPTR_017G011900v3	1251	262.51	23.84	-2.321	9.85×10^{-22}	Down	gi|743915676|ref|XP_011001798.1|/2.0e-166/PR× 10DICT× 10D: mannose/glucose-specific lectin-like isoform X3 [Populus euphratica]//gi|743915676|ref|XP_011001798.1|/3.6e-152/PR× 10DICT× 10D: mannose/glucose-specific lectin-like isoform X3 [Populus euphratica]//gi|743915676|ref|XP_011001798.1|/4.2e-157/PR× 10DICT× 10D: mannose/glucose-specific lectin-like isoform X3 [Populus euphratica]//gi|743915676|ref|XP_011001798.1|/6.1e-257/PR× 10DICT× 10D: mannose/glucose-specific lectin-like isoform X3 [Populus euphratica]//gi|743915676|ref|XP_011001798.1|/8.2e-165/PR× 10DICT× 10D: mannose/glucose-specific lectin-like isoform X3 [Populus euphratica]
POPTR_005G244100v3	1254	68.47	565.16	1.12164	9.57×10^{-6}	Up	gi|224085934|ref|XP_002307745.1|/1.4e-152/alpha-expansin 11 precursor family protein [Populus trichocarpa]
POPTR_013G100800v3	1417	154.37	36.71	-1.2853	3.18×10^{-7}	Down	gi|566200479|ref|XP_006376161.1|/1.5e-183/hypothetical protein POPTR_0013s10370g [Populus trichocarpa]//gi|566200479|ref|XP_006376161.1|/4.7e-153/hypothetical protein POPTR_0013s10370g [Populus trichocarpa]
POPTR_016G133900v3	2290	126.46	54.28	-1.0822	5.61×10^{-8}	Down	gi|224140583|ref|XP_002323661.1|/1.3e-248/S× 10RIN× 10/THR× 10ONIN× 10 protein KINAS× 10 1 [Populus trichocarpa]
POPTR_016G139400v3	1594	141.04	49.29	-1.2956	7.97×10^{-10}	Down	gi|566210302|ref|XP_006374054.1|/2.4e-284/hypothetical protein POPTR_0016s14620g, partial [Populus trichocarpa]
POPTR_001G173600v3	2537	2.37	41.94	1.82532	4.97×10^{-13}	Up	gi|566149557|ref|XP_002298111.2|/0.0e+00/cysteine-rich polycomb-like family protein [Populus trichocarpa]
POPTR_015G006200v3	1250	68.98	13.83	-1.5824	1.01×10^{-10}	Down	gi|566205296|ref|XP_002321965.2|/1.9e-221/hypothetical protein POPTR_0015s00860g [Populus trichocarpa]//gi|566205296|ref|XP_002321965.2|/3.8e-203/hypothetical protein POPTR_0015s00860g [Populus trichocarpa]//gi|566205296|ref|XP_002321965.2|/5.6e-151/hypothetical protein POPTR_0015s00860g [Populus trichocarpa]//gi|566205296|ref|XP_002321965.2|/7.5e-201/hypothetical protein POPTR_0015s00860g [Populus trichocarpa]
POPTR_011G122400v3	1597	504.04	1379.3	1.10574	2.03×10^{-9}	Up	gi|224117218|ref|XP_002317511.1|/5.5e-225/NADPH-protochlorophyllide oxidoreductase family protein [Populus trichocarpa]
POPTR_T115400v3	1020	252.39	114.05	-1.0167	2.18×10^{-7}	Down	gi|566185266|ref|XP_006380112.1|/2.5e-121/hypothetical protein POPTR_0008s22020g [Populus trichocarpa]
POPTR_009G033900v3	1455	52.38	204.45	1.11312	7.37×10^{-6}	Up	gi|566186162|ref|XP_002313425.2|/1.2e-219/hypothetical protein POPTR_0009s03900g [Populus trichocarpa]//gi|566186162|ref|XP_002313425.2|/1.5e-277/hypothetical protein POPTR_0009s03900g [Populus trichocarpa]//gi|566186162|ref|XP_002313425.2|/1.6e-279/hypothetical protein POPTR_0009s03900g [Populus trichocarpa]//gi|566186162|ref|XP_002313425.2|/3.0e-257/hypothetical protein POPTR_0009s03900g [Populus trichocarpa]
POPTR_003G118200v3	1733	22.25	66.82	1.03448	5.23×10^{-6}	Up	gi|566162216|ref|XP_002303546.2|/2.1e-290/hypothetical protein POPTR_0003s11810g [Populus trichocarpa]
POPTR_012G034400v3	604	41.4	145.95	1.43961	9.59×10^{-18}	Up	-
POPTR_006G093200v3	861	2.66	21.68	1.20938	2.04×10^{-6}	Up	gi|566175385|ref|XP_002309099.2|/3.5e-76/hypothetical protein POPTR_0006s09430g [Populus trichocarpa]
POPTR_011G048500v3	1179	6.62	49.97	1.73774	3.01×10^{-13}	Up	gi|566193740|ref|XP_002317298.2|/1.3e-156/hypothetical protein POPTR_0011s04830g [Populus trichocarpa]
POPTR_008G130000v3	1864	353.2	848.47	1.0664	2.50×10^{-18}	Up	gi|566183685|ref|XP_002312441.2|/8.1e-279/hypothetical protein POPTR_0008s12890g [Populus trichocarpa]
POPTR_014G071800v3	1776	142.65	364.14	1.01523	3.21×10^{-7}	Up	gi|566202964|ref|XP_002320070.2|/7.7e-255/hypothetical protein POPTR_0014s06750g [Populus trichocarpa]
POPTR_016G067700v3	873	5.83	29.07	1.12762	7.47×10^{-6}	Up	gi|566177461|ref|XP_002308331.2|/2.9e-114/hypothetical protein POPTR_0006s21590g [Populus trichocarpa]
POPTR_001G176100v3	1118	62.95	2.4	-1.35	7.01×10^{-9}	Down	gi|566149599|ref|XP_002298128.2|/4.4e-185/hypothetical protein POPTR_0001s17630g [Populus trichocarpa]
POPTR_019G086800v3	1692	311.62	1037.56	1.27616	4.13×10^{-10}	Up	gi|224145217|ref|XP_002325567.1|/1.7e-281/exostosin family protein [Populus trichocarpa]
POPTR_005G087000v3	600	47.32	167.91	1.38771	7.27×10^{-14}	Up	gi|566170281|ref|XP_006382935.1|/1.4e-36/hypothetical protein POPTR_0005s08920g [Populus trichocarpa]//gi|566170283|ref|XP_006382936.1|/4.8e-37/hypothetical protein POPTR_0005s08920g [Populus trichocarpa]
POPTR_001G015500v3	2876	213.6	72.35	-1.0806	8.38×10^{-6}	Down	gi|566147337|ref|XP_006368563.1|/0.0e+00/hypothetical protein POPTR_0001s05330g [Populus trichocarpa]//gi|566147339|ref|XP_006368564.1|/0.0e+00/hypothetical protein POPTR_0001s05330g [Populus trichocarpa]

<table>
<tr><td>POPTR_T051200v3</td><td>2117</td><td>50.82</td><td>17.32</td><td>-1.1945</td><td>5.59×10^{-7}</td><td>Down</td><td>gi|224145766|ref|XP_002325757.1|/6.7e-111/hypothetical protein POPTR_0019s03110g [Populus trichocarpa]//gi|566258644|ref|XP_006388901.1|/2.8e-224/hypothetical protein POPTR_0078s00210g [Populus trichocarpa]</td></tr>
<tr><td>POPTR_002G125000v3</td><td>786</td><td>24.97</td><td>132.1</td><td>1.8277</td><td>1.25×10^{-21}</td><td>Up</td><td>gi|566157687|ref|XP_002301178.2|/2.9e-142/basic helix-loop-helix family protein [Populus trichocarpa]</td></tr>
<tr><td>POPTR_002G067600v3</td><td>1482</td><td>107.22</td><td>290.45</td><td>1.15751</td><td>4.76×10^{-13}</td><td>Up</td><td>gi|566156621|ref|XP_002300916.2|/3.2e-122/hypothetical protein POPTR_0002s06820g [Populus trichocarpa]</td></tr>
<tr><td>POPTR_017G037900v3</td><td>1428</td><td>173.13</td><td>82.27</td><td>-1.0593</td><td>3.63×10^{-10}</td><td>Down</td><td>gi|566211808|ref|XP_006372956.1|/2.9e-162/hypothetical protein POPTR_0017s06550g [Populus trichocarpa]//gi|566211810|ref|XP_006372957.1|/1.4e-186/hypothetical protein POPTR_0017s06550g [Populus trichocarpa]</td></tr>
<tr><td>POPTR_009G142000v3</td><td>1291</td><td>166.51</td><td>25.44</td><td>-1.8838</td><td>5.24×10^{-15}</td><td>Down</td><td>gi|81593|pir||S18750/1.0e-192/chitinase (× 10C 3.2.1.14) precursor - western balsam poplar x cottonwood</td></tr>
<tr><td>POPTR_010G151200v3</td><td>654</td><td>64.6</td><td>1.7</td><td>-1.6526</td><td>8.11×10^{-12}</td><td>Down</td><td>gi|224108824|ref|XP_002314981.1|/5.1e-56/pop3 peptide family protein [Populus trichocarpa]</td></tr>
<tr><td>POPTR_017G046200v3</td><td>2937</td><td>472.35</td><td>149.11</td><td>-1.4848</td><td>1.12×10^{-15}</td><td>Down</td><td>gi|566147339|ref|XP_006368564.1|/0.0e+00/hypothetical protein POPTR_0001s05330g [Populus trichocarpa]//gi|566211990|ref|XP_002323952.2|/0.0e+00/hypothetical protein POPTR_0017s07350g [Populus trichocarpa]</td></tr>
<tr><td>POPTR_009G142100v3</td><td>1172</td><td>67.11</td><td>7.5</td><td>-1.5535</td><td>9.34×10^{-10}</td><td>Down</td><td>gi|566187967|ref|XP_006379300.1|/2.2e-192/hypothetical protein POPTR_0009s14410g, partial [Populus trichocarpa]</td></tr>
<tr><td>POPTR_003G094700v3</td><td>2148</td><td>57.89</td><td>23</td><td>-1.0327</td><td>4.34×10^{-6}</td><td>Down</td><td>gi|566161765|ref|XP_002304348.2|/0.0e+00/hypothetical protein POPTR_0003s09340g [Populus trichocarpa]</td></tr>
<tr><td>POPTR_009G164500v3</td><td>1275</td><td>36.38</td><td>140.43</td><td>1.34531</td><td>1.42×10^{-9}</td><td>Up</td><td>gi|224102821|ref|XP_002312814.1|/2.1e-177/basic leucine zipper transcription factor family protein [Populus trichocarpa]//gi|224102821|ref|XP_002312814.1|/2.5e-189/basic leucine zipper transcription factor family protein [Populus trichocarpa]</td></tr>
<tr><td>POPTR_005G232000v3</td><td>2650</td><td>264.46</td><td>796.33</td><td>1.34909</td><td>9.54×10^{-21}</td><td>Up</td><td>gi|566173177|ref|XP_002306891.2|/5.4e-245/hypothetical protein POPTR_0005s25350g [Populus trichocarpa]//gi|566173177|ref|XP_002306891.2|/7.5e-242/hypothetical protein POPTR_0005s25350g [Populus trichocarpa]</td></tr>
<tr><td>POPTR_015G111100v3</td><td>932</td><td>10.24</td><td>118.93</td><td>1.9541</td><td>1.01×10^{-15}</td><td>Up</td><td>gi|566207215|ref|XP_006374588.1|/7.5e-143/hypothetical protein POPTR_0015s12260g [Populus trichocarpa]</td></tr>
<tr><td>POPTR_002G207400v3</td><td>1594</td><td>50.02</td><td>15.75</td><td>-1.2652</td><td>5.63×10^{-8}</td><td>Down</td><td>gi|566260224|ref|XP_006389665.1|/3.3e-163/hypothetical protein POPTR_0020s00240g [Populus trichocarpa]//gi|566260224|ref|XP_006389665.1|/7.6e-224/hypothetical protein POPTR_0020s00240g [Populus trichocarpa]</td></tr>
<tr><td>POPTR_013G100500v3</td><td>1292</td><td>10.72</td><td>46.51</td><td>1.13169</td><td>4.86×10^{-6}</td><td>Up</td><td>gi|566200487|ref|XP_006376165.1|/1.5e-157/hypothetical protein POPTR_0013s10400g [Populus trichocarpa]</td></tr>
<tr><td>POPTR_006G136700v3</td><td>4017</td><td>1537.66</td><td>608.35</td><td>-1.1234</td><td>9.40×10^{-8}</td><td>Down</td><td>gi|566176273|ref|XP_006381564.1|/0.0e+00/sucrose synthase family protein [Populus trichocarpa]</td></tr>
<tr><td>POPTR_006G030300v3</td><td>1356</td><td>75.96</td><td>456.42</td><td>1.8763</td><td>1.41×10^{-19}</td><td>Up</td><td>gi|566174172|ref|XP_002307943.2|/5.7e-202/hypothetical protein POPTR_0006s02890g [Populus trichocarpa]</td></tr>
<tr><td>POPTR_005G122600v3</td><td>1231</td><td>68.12</td><td>15.76</td><td>-1.2286</td><td>1.25×10^{-6}</td><td>Down</td><td>gi|566171010|ref|XP_006383191.1|/5.2e-178/hydrolase family protein [Populus trichocarpa]//gi|566171010|ref|XP_006383191.1|/7.3e-180/hydrolase family protein [Populus trichocarpa]//gi|743919419|ref|XP_011003733.1|/3.1e-140/PR× 10DICT× 10D: epoxide hydrolase 3-like isoform X2 [Populus euphratica]//gi|743919419|ref|XP_011003733.1|/3.8e-145/PR× 10DICT× 10D: epoxide hydrolase 3-like isoform X2 [Populus euphratica]</td></tr>
<tr><td>POPTR_015G026500v3</td><td>1588</td><td>24.53</td><td>2.57</td><td>-1.1607</td><td>1.70×10^{-6}</td><td>Down</td><td>gi|566206063|ref|XP_006374295.1|/2.0e-238/hypothetical protein POPTR_0015s05760g [Populus trichocarpa]</td></tr>
<tr><td>POPTR_001G450600v3</td><td>2396</td><td>134.82</td><td>60.16</td><td>-1.0462</td><td>2.42×10^{-7}</td><td>Down</td><td>gi|566155058|ref|XP_002298975.2|/0.0e+00/hypothetical protein POPTR_0001s45490g [Populus trichocarpa]</td></tr>
<tr><td>POPTR_003G193200v3</td><td>1231</td><td>430.91</td><td>1599.67</td><td>1.49633</td><td>3.29×10^{-17}</td><td>Up</td><td>gi|566163522|ref|XP_002303948.2|/2.1e-190/hypothetical protein POPTR_0003s19260g [Populus trichocarpa]</td></tr>
<tr><td>POPTR_003G177200v3</td><td>1794</td><td>14.6</td><td>63.99</td><td>1.04171</td><td>3.97×10^{-5}</td><td>Up</td><td>gi|224075607|ref|XP_002304704.1|/4.0e-83/hypothetical protein POPTR_0003s17570g [Populus trichocarpa]//gi|224075607|ref|XP_002304704.1|/7.0e-42/hypothetical protein POPTR_0003s17570g [Populus trichocarpa]</td></tr>
<tr><td>POPTR_001G083600v3</td><td>1839</td><td>273.45</td><td>88.36</td><td>-1.2969</td><td>8.18×10^{-9}</td><td>Down</td><td>gi|566148398|ref|XP_002299426.2|/0.0e+00/hypothetical protein POPTR_0001s11390g, partial [Populus trichocarpa]</td></tr>
<tr><td>POPTR_011G058400v3</td><td>1503</td><td>17.15</td><td>75.99</td><td>1.22854</td><td>3.86×10^{-7}</td><td>Up</td><td>gi|566193949|ref|XP_006377419.1|/1.7e-181/hypothetical protein POPTR_0011s05740g [Populus trichocarpa]//gi|566193949|ref|XP_006377419.1|/3.0e-198/hypothetical protein POPTR_0011s05740g [Populus trichocarpa]</td></tr>
<tr><td>POPTR_008G072400v3</td><td>1972</td><td>19.74</td><td>62.88</td><td>1.0275</td><td>1.20×10^{-5}</td><td>Up</td><td>gi|566182788|ref|XP_002311245.2|/0.0e+00/hypothetical protein POPTR_0008s07240g [Populus trichocarpa]</td></tr>
<tr><td>POPTR_011G044000v3</td><td>1342</td><td>129.8</td><td>50.7</td><td>-1.141</td><td>8.42×10^{-8}</td><td>Down</td><td>gi|224114045|ref|XP_002316652.1|/1.3e-167/hypothetical protein POPTR_0011s04380g [Populus trichocarpa]//gi|566193658|ref|XP_006377303.1|/2.4e-183/hypothetical protein POPTR_0011s04380g [Populus trichocarpa]</td></tr>
<tr><td>POPTR_006G115200v3</td><td>1745</td><td>50.16</td><td>239</td><td>1.71879</td><td>3.04×10^{-19}</td><td>Up</td><td>gi|566175751|ref|XP_006381307.1|/3.0e-220/hypothetical protein POPTR_0006s11630g [Populus trichocarpa]</td></tr>
<tr><td>POPTR_018G104500v3</td><td>648</td><td>26.93</td><td>74.64</td><td>1.037</td><td>6.95×10^{-7}</td><td>Up</td><td>gi|224142365|ref|XP_002324529.1|/2.7e-61/hypothetical protein POPTR_0018s11360g [Populus trichocarpa]</td></tr>
<tr><td>POPTR_003G061600v3</td><td>1570</td><td>26.89</td><td>81.89</td><td>1.13262</td><td>8.56×10^{-8}</td><td>Up</td><td>gi|743893576|ref|XP_011040025.1|/1.7e-159/PR× 10DICT× 10D: zinc finger CCCH domain-containing protein 39-like [Populus euphratica]</td></tr>
<tr><td>POPTR_004G023800v3</td><td>3114</td><td>52.92</td><td>8.66</td><td>-1.3524</td><td>1.06×10^{-7}</td><td>Down</td><td>gi|743926957|ref|XP_011007652.1|/0.0e+00/PR× 10DICT× 10D: cysteine-rich receptor-like protein kinase 10 [Populus euphratica]</td></tr>
<tr><td>POPTR_010G163100v3</td><td>1542</td><td>19.51</td><td>3.99</td><td>-1.1628</td><td>4.86×10^{-6}</td><td>Down</td><td>gi|566191258|ref|XP_002316106.2|/8.7e-259/hypothetical protein POPTR_0010s17050g [Populus trichocarpa]//gi|566191258|ref|XP_002316106.2|/9.7e-307/hypothetical</td></tr>
</table>

<table>
<tr><td></td><td></td><td></td><td></td><td></td><td></td><td></td><td>protein POPTR_0010s17050g [Populus trichocarpa]</td></tr>
<tr><td>POPTR_007G096200v3</td><td>1337</td><td>95.64</td><td>489.19</td><td>1.6781</td><td>9.51×10^{-15}</td><td>Up</td><td>gi|224096149|ref|XP_002310551.1|/3.8e-187/Peroxidase 17 precursor family protein [Populus trichocarpa]</td></tr>
<tr><td>POPTR_001G117000v3</td><td>1585</td><td>254.22</td><td>617.28</td><td>1.02725</td><td>2.23×10^{-13}</td><td>Up</td><td>gi|566147855|ref|XP_006368711.1|/7.0e-254/hypothetical protein POPTR_0001s08010g [Populus trichocarpa]</td></tr>
<tr><td>POPTR_008G178300v3</td><td>1455</td><td>91.52</td><td>241.95</td><td>1.05033</td><td>5.08×10^{-8}</td><td>Up</td><td>gi|743853901|ref|XP_011029650.1|/6.0e-141/PR× 10DICT× 10D: uncharacterized protein At3g61260 [Populus euphratica]</td></tr>
<tr><td>POPTR_010G112900v3</td><td>1072</td><td>25.72</td><td>116.72</td><td>1.21838</td><td>6.96×10^{-7}</td><td>Up</td><td>gi|224108337|ref|XP_002314811.1|/3.5e-143/hypothetical protein POPTR_0010s12330g [Populus trichocarpa]</td></tr>
<tr><td>POPTR_014G145100v3</td><td>1491</td><td>402.1</td><td>127.19</td><td>-1.0117</td><td>5.62×10^{-5}</td><td>Down</td><td>gi|224131424|ref|XP_002321081.1|/2.1e-224/chalcone synthase family protein [Populus trichocarpa]//gi|224131424|ref|XP_002321081.1|/5.0e-226/chalcone synthase family protein [Populus trichocarpa]//gi|224131424|ref|XP_002321081.1|/9.1e-228/chalcone synthase family protein [Populus trichocarpa]</td></tr>
<tr><td>POPTR_017G141400v3</td><td>2294</td><td>63.92</td><td>264.11</td><td>1.01786</td><td>5.86×10^{-5}</td><td>Up</td><td>gi|566210762|ref|XP_006372459.1|/0.0e+00/hypothetical protein POPTR_0017s01830g [Populus trichocarpa]//gi|566210762|ref|XP_006372459.1|/6.1e-310/hypothetical protein POPTR_0017s01830g [Populus trichocarpa]</td></tr>
<tr><td>POPTR_003G068900v3</td><td>1387</td><td>94.33</td><td>25.73</td><td>-1.0669</td><td>2.75×10^{-5}</td><td>Down</td><td>gi|224073600|ref|XP_002304118.1|/1.6e-143/hypothetical protein POPTR_0003s06670g [Populus trichocarpa]//gi|224073600|ref|XP_002304118.1|/9.6e-118/hypothetical protein POPTR_0003s06670g [Populus trichocarpa]</td></tr>
<tr><td>POPTR_003G127500v3</td><td>3391</td><td>277.38</td><td>764.33</td><td>1.11014</td><td>9.72×10^{-10}</td><td>Up</td><td>gi|566162357|ref|XP_002303590.2|/0.0e+00/hypothetical protein POPTR_0003s12760g [Populus trichocarpa]</td></tr>
<tr><td>POPTR_010G059200v3</td><td>1977</td><td>47.41</td><td>120.32</td><td>1.01479</td><td>2.58×10^{-8}</td><td>Up</td><td>gi|224107619|ref|XP_002314539.1|/1.2e-203/hypothetical protein POPTR_0010s06910g [Populus trichocarpa]</td></tr>
<tr><td>POPTR_006G232700v3</td><td>1540</td><td>24.38</td><td>100.87</td><td>1.20607</td><td>5.03×10^{-7}</td><td>Up</td><td>gi|224088913|ref|XP_002308579.1|/5.2e-192/hypothetical protein POPTR_0006s24880g [Populus trichocarpa]</td></tr>
<tr><td>POPTR_007G103800v3</td><td>1626</td><td>24.4</td><td>0.89</td><td>-1.4936</td><td>6.04×10^{-10}</td><td>Down</td><td>gi|224096043|ref|XP_002310523.1|/1.1e-225/gibberellin 20-oxidase family protein [Populus trichocarpa]//gi|224096043|ref|XP_002310523.1|/4.5e-171/gibberellin 20-oxidase family protein [Populus trichocarpa]</td></tr>
<tr><td>POPTR_011G049000v3</td><td>1016</td><td>13.54</td><td>116.12</td><td>1.95983</td><td>4.47×10^{-17}</td><td>Up</td><td>gi|566193750|ref|XP_006377321.1|/4.6e-165/hypothetical protein POPTR_0011s04870g [Populus trichocarpa]</td></tr>
<tr><td>POPTR_019G107600v3</td><td>1639</td><td>7.75</td><td>70.59</td><td>1.07812</td><td>1.85×10^{-5}</td><td>Up</td><td>gi|743892859|ref|XP_011039779.1|/3.7e-207/PR× 10DICT× 10D: ankyrin repeat-containing protein At5g02620-like isoform X2 [Populus euphratica]</td></tr>
<tr><td>POPTR_013G020900v3</td><td>3205</td><td>54.64</td><td>177.71</td><td>1.06454</td><td>5.58×10^{-6}</td><td>Up</td><td>gi|566198944|ref|XP_002319536.2|/0.0e+00/resistance family protein [Populus trichocarpa]</td></tr>
<tr><td>POPTR_T012500v3</td><td>1302</td><td>7062.12</td><td>23675.1</td><td>1.22143</td><td>1.36×10^{-8}</td><td>Up</td><td>gi|224144484|ref|XP_002325305.1|/7.6e-178/embryonic abundant family protein [Populus trichocarpa]</td></tr>
<tr><td>POPTR_005G230200v3</td><td>1824</td><td>53.68</td><td>345.65</td><td>2.11318</td><td>1.90×10^{-27}</td><td>Up</td><td>gi|224082898|ref|XP_002306883.1|/3.4e-252/hypothetical protein POPTR_0005s25180g [Populus trichocarpa]</td></tr>
<tr><td>POPTR_004G030800v3</td><td>1261</td><td>93.06</td><td>281.85</td><td>1.08424</td><td>9.27×10^{-7}</td><td>Up</td><td>gi|224078820|ref|XP_002305640.1|/2.7e-224/hypothetical protein POPTR_0004s03040g [Populus trichocarpa]//gi|743918764|ref|XP_011003391.1|/6.0e-227/PR× 10DICT× 10D: uncharacterized protein LOC105110150 [Populus euphratica]</td></tr>
<tr><td>POPTR_007G007100v3</td><td>2406</td><td>294.84</td><td>6908.4</td><td>3.46021</td><td>2.35×10^{-66}</td><td>Up</td><td>gi|566181359|ref|XP_002310328.2|/0.0e+00/hypothetical protein POPTR_0007s14720g [Populus trichocarpa]</td></tr>
<tr><td>POPTR_001G191800v3</td><td>2116</td><td>71.86</td><td>226.48</td><td>1.02943</td><td>1.41×10^{-5}</td><td>Up</td><td>gi|743935373|ref|XP_011012055.1|/3.0e-240/PR× 10DICT× 10D: transcription factor bHLH87 [Populus euphratica]</td></tr>
<tr><td>POPTR_005G023900v3</td><td>1621</td><td>53.1</td><td>330.87</td><td>1.33297</td><td>1.20×10^{-7}</td><td>Up</td><td>gi|566168965|ref|XP_006382458.1|/1.8e-116/nodulin MtN3 family protein [Populus trichocarpa]//gi|566168965|ref|XP_006382458.1|/1.9e-96/nodulin MtN3 family protein [Populus trichocarpa]//gi|743882929|ref|XP_011036889.1|/2.6e-121/PR× 10DICT× 10D: bidirectional sugar transporter SW× 10× 10T16-like isoform X2 [Populus euphratica]</td></tr>
<tr><td>POPTR_016G038000v3</td><td>1522</td><td>143.05</td><td>34.73</td><td>-1.149</td><td>6.42×10^{-6}</td><td>Down</td><td>gi|224137712|ref|XP_002322625.1|/1.2e-60/hypothetical protein POPTR_0016s03750g [Populus trichocarpa]//gi|224137712|ref|XP_002322625.1|/1.8e-75/hypothetical protein POPTR_0016s03750g [Populus trichocarpa]//gi|224137712|ref|XP_002322625.1|/1.8e-95/hypothetical protein POPTR_0016s03750g [Populus trichocarpa]</td></tr>
<tr><td>POPTR_012G124100v3</td><td>3528</td><td>21.97</td><td>86.1</td><td>1.25975</td><td>5.59×10^{-8}</td><td>Up</td><td>gi|566198544|ref|XP_002318300.2|/8.7e-270/hypothetical protein POPTR_0012s14920g [Populus trichocarpa]</td></tr>
<tr><td>POPTR_007G074700v3</td><td>1025</td><td>26.87</td><td>98.35</td><td>1.12159</td><td>3.17×10^{-6}</td><td>Up</td><td>gi|566180058|ref|XP_002310058.2|/4.6e-193/hypothetical protein POPTR_0007s07330g [Populus trichocarpa]</td></tr>
<tr><td>POPTR_006G204200v3</td><td>1120</td><td>121.29</td><td>38.63</td><td>-1.2519</td><td>7.57×10^{-8}</td><td>Down</td><td>gi|566177532|ref|XP_006381955.1|/8.5e-116/hypothetical protein POPTR_0006s22060g [Populus trichocarpa]</td></tr>
<tr><td>POPTR_002G197300v3</td><td>2298</td><td>734.89</td><td>1726.02</td><td>1.02683</td><td>6.67×10^{-18}</td><td>Up</td><td>gi|566158998|ref|XP_002302757.2|/4.1e-164/DNAJ heat shock N-terminal domain-containing family protein [Populus trichocarpa]//gi|566158998|ref|XP_002302757.2|/6.8e-261/DNAJ heat shock N-terminal domain-containing family protein [Populus trichocarpa]</td></tr>
<tr><td>POPTR_013G101000v3</td><td>1495</td><td>99.42</td><td>15.42</td><td>-1.3908</td><td>4.47×10^{-8}</td><td>Down</td><td>gi|566200475|ref|XP_006376159.1|/2.5e-183/hypothetical protein POPTR_0013s10350g [Populus trichocarpa]</td></tr>
<tr><td>POPTR_005G175200v3</td><td>1253</td><td>27.85</td><td>172.99</td><td>1.88496</td><td>1.72×10^{-18}</td><td>Up</td><td>gi|743915000|ref|XP_011001442.1|/3.1e-184/PR× 10DICT× 10D: chalcone synthase-like [Populus euphratica]</td></tr>
<tr><td>POPTR_018G015100v3</td><td>1836</td><td>105.17</td><td>45.92</td><td>-1.002</td><td>2.57×10^{-6}</td><td>Down</td><td>gi|224142001|ref|XP_002324349.1|/4.6e-274/nucleoid DNA-binding family protein [Populus trichocarpa]</td></tr>
<tr><td>POPTR_006G183200v3</td><td>2122</td><td>198.36</td><td>545.69</td><td>1.19885</td><td>1.47×10^{-17}</td><td>Up</td><td>gi|224088300|ref|XP_002308408.1|/4.1e-250/BRI1 SUPPR× 10SSOR 1 family protein [Populus trichocarpa]//gi|224088300|ref|XP_002308408.1|/5.1e-275/BRI1 SUPPR× 10SSOR 1 family protein [Populus trichocarpa]</td></tr>
<tr><td>POPTR_008G043900v3</td><td>1307</td><td>25.84</td><td>1.78</td><td>-1.0629</td><td>3.97×10^{-6}</td><td>Down</td><td>gi|566182312|ref|XP_002312035.2|/7.6e-83/hypothetical protein POPTR_0008s04350g [Populus trichocarpa]</td></tr>
</table>

POPTR_009G159700v3	2163	61.92	172.8	1.11308	8.89×10^{-9}	Up	gi|566188280|ref|XP_002312838.2|/0.0e+00/ascorbate oxidase precursor family protein [Populus trichocarpa]//gi|566188280|ref|XP_002312838.2|/1.7e-275/ascorbate oxidase precursor family protein [Populus trichocarpa]
POPTR_002G232500v3	1280	28.9	4.32	-1.2564	6.06×10^{-7}	Down	gi|566159580|ref|XP_002302890.2|/4.3e-77/hypothetical protein POPTR_0002s23350g, partial [Populus trichocarpa]
POPTR_005G028000v3	1529	167.53	545.98	1.4023	4.77×10^{-17}	Up	gi|566169067|ref|XP_006382508.1|/1.0e-264/hypothetical protein POPTR_0005s02810g [Populus trichocarpa]
POPTR_001G094700v3	785	116.28	569.54	1.49612	6.94×10^{-11}	Up	gi|224053805|ref|XP_002297988.1|/1.7e-09/hypothetical protein POPTR_0001s10310g [Populus trichocarpa]
POPTR_016G057400v3	1340	268.97	77.57	-1.2578	1.37×10^{-7}	Down	gi|566208909|ref|XP_006373789.1|/2.3e-187/hypothetical protein POPTR_0016s05780g [Populus trichocarpa]
POPTR_014G116800v3	1192	17.68	59.7	1.08953	3.91×10^{-6}	Up	gi|566203730|ref|XP_002320958.2|/4.5e-155/putative galactinol synthase family protein [Populus trichocarpa]//gi|566203730|ref|XP_002320958.2|/5.1e-127/putative galactinol synthase family protein [Populus trichocarpa]//gi|566203730|ref|XP_002320958.2|/5.7e-199/putative galactinol synthase family protein [Populus trichocarpa]
POPTR_008G131100v3	744	413.96	1210.94	1.20132	7.25×10^{-12}	Up	gi|224099501|ref|XP_002311508.1|/4.0e-83/putative major latex family protein [Populus trichocarpa]
POPTR_015G098400v3	775	674.7	217.98	-1.6138	2.57×10^{-28}	Down	gi|157101710|gb|ABV23568.1|/1.7e-40/MADS-box protein [Populus deltoides]//gi|224133998|ref|XP_002321711.1|/1.5e-78/putative transcription factor family protein [Populus trichocarpa]//gi|224133998|ref|XP_002321711.1|/2.2e-100/putative transcription factor family protein [Populus trichocarpa]//gi|224133998|ref|XP_002321711.1|/2.6e-95/putative transcription factor family protein [Populus trichocarpa]//gi|224133998|ref|XP_002321711.1|/2.7e-95/putative transcription factor family protein [Populus trichocarpa]//gi|224133998|ref|XP_002321711.1|/9.3e-66/putative transcription factor family protein [Populus trichocarpa]//gi|743831182|ref|XP_011023983.1|/1.9e-84/PR× 10DICT× 10D: truncated transcription factor CAULIFLOW× 10R D-like isoform X1 [Populus euphratica]//gi|743831182|ref|XP_011023983.1|/5.9e-76/PR× 10DICT× 10D: truncated transcription factor CAULIFLOW× 10R D-like isoform X1 [Populus euphratica]
POPTR_013G067000v3	2364	18.38	85.65	1.56968	9.83×10^{-13}	Up	gi|224126005|ref|XP_002319732.1|/1.7e-226/hypothetical protein POPTR_0013s06320g [Populus trichocarpa]
POPTR_001G079800v3	1158	175.2	72.69	-1.009	1.19×10^{-5}	Down	gi|224057956|ref|XP_002299408.1|/1.6e-157/ethylene-responsive element-binding family protein [Populus trichocarpa]
POPTR_016G015200v3	773	300.49	797.44	1.13203	1.02×10^{-14}	Up	gi|566208155|ref|XP_002323163.2|/3.6e-91/hypothetical protein POPTR_0016s01690g [Populus trichocarpa]//gi|566208155|ref|XP_002323163.2|/7.4e-90/hypothetical protein POPTR_0016s01690g [Populus trichocarpa]
POPTR_001G015600v3	3010	1125.05	345.71	-1.4831	2.58×10^{-14}	Down	gi|566256300|ref|XP_006388115.1|/0.0e+00/hypothetical protein POPTR_0328s00200g [Populus trichocarpa]
POPTR_006G240400v3	1665	74.13	214.75	1.2903	2.35×10^{-22}	Up	gi|566178147|ref|XP_002308611.2|/3.1e-155/hypothetical protein POPTR_0006s25710g [Populus trichocarpa]
POPTR_T055600v3	1248	24.73	3.07	-1.3328	1.41×10^{-7}	Down	gi|566230311|ref|XP_006371128.1|/1.1e-112/hypothetical protein POPTR_0019s04390g [Populus trichocarpa]
POPTR_019G079200v3	2283	19.4	70.57	1.09374	6.27×10^{-6}	Up	gi|566240129|ref|XP_006371457.1|/0.0e+00/hypothetical protein POPTR_0019s10800g [Populus trichocarpa]
POPTR_010G209900v3	915	571.27	159.69	-1.2811	1.31×10^{-7}	Down	gi|224112847|ref|XP_002316308.1|/1.7e-93/Gamma-tonoplast intrinsic protein 3 [Populus trichocarpa]//gi|224112847|ref|XP_002316308.1|/9.5e-138/Gamma-tonoplast intrinsic protein 3 [Populus trichocarpa]
POPTR_001G185700v3	2199	1031.67	2379.26	1.00323	7.80×10^{-15}	Up	gi|566149767|ref|XP_002299719.2|/0.0e+00/hypothetical protein POPTR_0001s18640g [Populus trichocarpa]
POPTR_015G103200v3	755	1.29	21.89	1.34041	1.33×10^{-7}	Up	-
POPTR_015G133600v3	522	25.42	80.75	1.12629	1.07×10^{-7}	Up	gi|566207528|ref|XP_006374648.1|/1.3e-62/hypothetical protein POPTR_0015s14130g, partial [Populus trichocarpa]
POPTR_019G024600v3	1475	329.24	13.56	-3.2385	1.24×10^{-42}	Down	gi|224144643|ref|XP_002325360.1|/9.3e-211/hypothetical protein POPTR_0019s04130g [Populus trichocarpa]
POPTR_001G049700v3	1201	50.12	149.21	1.27774	2.45×10^{-16}	Up	gi|224057575|ref|XP_002299275.1|/7.9e-46/hypothetical protein POPTR_0001s14510g [Populus trichocarpa]
POPTR_T143400v3	1514	10.4	62.78	1.18411	3.17×10^{-6}	Up	gi|566165054|ref|XP_002305755.2|/2.3e-220/pyruvate decarboxylase family protein [Populus trichocarpa]
POPTR_001G169000v3	1317	255.46	115.77	-1.0225	2.69×10^{-6}	Down	gi|566149485|ref|XP_002299591.2|/1.6e-111/germin-like family protein [Populus trichocarpa]
POPTR_001G448100v3	1735	52.22	233.42	1.46856	4.07×10^{-11}	Up	gi|566155005|ref|XP_002300498.2|/2.1e-252/hypothetical protein POPTR_0001s45220g [Populus trichocarpa]
POPTR_014G069500v3	1254	14.2	64.6	1.15616	4.20×10^{-6}	Up	gi|566202934|ref|XP_002320723.2|/6.4e-181/hypothetical protein POPTR_0014s06510g [Populus trichocarpa]
POPTR_004G096000v3	886	62.19	26.13	-1.0182	4.15×10^{-6}	Down	gi|224079367|ref|XP_002305838.1|/1.6e-37/hypothetical protein POPTR_0004s09510g [Populus trichocarpa]
POPTR_008G214600v3	918	62.89	9.6	-1.1526	2.66×10^{-6}	Down	gi|566185446|ref|XP_006380202.1|/6.4e-53/hypothetical protein POPTR_0008s22870g [Populus trichocarpa]
POPTR_006G027800v3	1246	395.2	1694.49	1.47154	1.36×10^{-11}	Up	gi|566174166|ref|XP_006380953.1|/1.4e-195/hypothetical protein POPTR_0006s02855g [Populus trichocarpa]
POPTR_009G074600v3	651	3.3	24.31	1.33667	1.26×10^{-7}	Up	-
POPTR_003G194200v3	2975	2.92	34.47	1.68988	1.45×10^{-11}	Up	gi|566163536|ref|XP_002303941.2|/0.0e+00/cation/proton exchanger family protein [Populus trichocarpa]
POPTR_007G021300v3	1587	34.16	2.94	-1.6432	9.86×10^{-11}	Down	gi|118486393|gb|ABK95036.1|/1.8e-156/unknown [Populus trichocarpa]//gi|118486393|gb|ABK95036.1|/7.8e-210/unknown [Populus trichocarpa]
POPTR_004G234900v3	2369	2	30.01	1.28835	3.74×10^{-7}	Up	gi|566168448|ref|XP_006385149.1|/0.0e+00/alcohol oxidase-related family protein [Populus trichocarpa]

POPTR_004G190100v3	2116	19.08	248.54	2.24345	5.91×10^{-21}	Up	gi∣566167513∣ref∣XP_006384683.1∣/0.0e+00/lecithin:cholesterol acyltransferase family protein [Populus trichocarpa]
POPTR_001G240600v3	1667	705.12	277.57	-1.2573	1.68×10^{-12}	Down	gi∣566150807∣ref∣XP_006369525.1∣/7.1e-261/hypothetical protein POPTR_0001s24740g [Populus trichocarpa]
POPTR_012G091500v3	1617	406.15	101.75	-1.4995	1.78×10^{-10}	Down	gi∣224121874∣ref∣XP_002318694.1∣/6.8e-231/pectate lyase 22 precursor family protein [Populus trichocarpa]
POPTR_019G093800v3	1163	88.65	13.22	-1.053	1.81×10^{-5}	Down	gi∣566242189∣ref∣XP_002326040.2∣/3.8e-164/hypothetical protein POPTR_0019s12360g [Populus trichocarpa]
POPTR_012G047700v3	1751	84.25	399.63	1.61247	2.10×10^{-14}	Up	gi∣566196787∣ref∣XP_002317877.2∣/2.2e-218/hypothetical protein POPTR_0012s04510g [Populus trichocarpa]
POPTR_016G031400v3	1591	3.33	33.01	1.71445	4.44×10^{-12}	Up	gi∣566208414∣ref∣XP_002323220.2∣/2.4e-220/hypothetical protein POPTR_0016s03120g [Populus trichocarpa]
POPTR_008G104700v3	1210	3.65	20.99	1.23817	8.82×10^{-7}	Up	gi∣566183280∣ref∣XP_002312327.2∣/2.4e-214/hypothetical protein POPTR_0008s10430g [Populus trichocarpa]//gi∣566183282∣ref∣XP_006379706.1∣/2.7e-210/hypothetical protein POPTR_0008s10430g [Populus trichocarpa]
POPTR_005G002100v3	329	17.92	62.48	1.1342	1.06×10^{-6}	Up	gi∣566168543∣ref∣XP_006382256.1∣/8.4e-23/hypothetical protein POPTR_0005s00390g [Populus trichocarpa]
POPTR_001G296500v3	1515	228.33	613.43	1.19487	1.11×10^{-19}	Up	gi∣224056230∣ref∣XP_002298766.1∣/2.4e-44/putative leucine-rich repeat family protein [Populus trichocarpa]//gi∣224056230∣ref∣XP_002298766.1∣/2.9e-55/putative leucine-rich repeat family protein [Populus trichocarpa]
POPTR_002G082200v3	1288	116.48	37.6	-1.1091	7.89×10^{-6}	Down	gi∣566156895∣ref∣XP_002302230.2∣/1.1e-98/hypothetical protein POPTR_0002s08270g [Populus trichocarpa]
POPTR_002G227800v3	471	447.36	1654.4	1.19319	1.40×10^{-7}	Up	gi∣566159231∣ref∣XP_006386776.1∣/1.4e-79/hypothetical protein POPTR_0002s21380g [Populus trichocarpa]
POPTR_019G063700v3	1687	21.09	5.81	-1.0109	7.09×10^{-5}	Down	gi∣566238140∣ref∣XP_006371337.1∣/1.1e-233/clathrin assembly family protein [Populus trichocarpa]
POPTR_005G058200v3	1287	92.59	261.17	1.15004	1.47×10^{-10}	Up	gi∣566169745∣ref∣XP_006382839.1∣/3.5e-137/hypothetical protein POPTR_0005s05950g [Populus trichocarpa]//gi∣566169747∣ref∣XP_006382840.1∣/7.0e-138/hypothetical protein POPTR_0005s05950g [Populus trichocarpa]
POPTR_018G021900v3	1262	86.67	256.3	1.10602	1.13×10^{-7}	Up	gi∣224141937∣ref∣XP_002324317.1∣/3.1e-104/hypothetical protein POPTR_0018s02280g [Populus trichocarpa]
POPTR_004G080900v3	3473	39.29	178.06	1.70721	3.17×10^{-19}	Up	gi∣743901121∣ref∣XP_011043875.1∣/0.0e+00/PR× 10DICT× 10D: uncharacterized protein LOC105139209 [Populus euphratica]
POPTR_004G073400v3	1089	0.35	15.93	1.35256	8.54×10^{-8}	Up	gi∣566165378∣ref∣XP_002305264.2∣/7.4e-168/hypothetical protein POPTR_0004s07170g [Populus trichocarpa]
POPTR_016G117100v3	1511	581.97	175.62	-1.5777	1.72×10^{-20}	Down	gi∣224140423∣ref∣XP_002323582.1∣/4.6e-168/hypothetical protein POPTR_0016s12440g [Populus trichocarpa]//gi∣224140423∣ref∣XP_002323582.1∣/5.8e-221/hypothetical protein POPTR_0016s12440g [Populus trichocarpa]
POPTR_007G044600v3	720	4.88	28.54	1.35904	4.01×10^{-8}	Up	gi∣224094310∣ref∣XP_002310136.1∣/3.0e-133/hypothetical protein POPTR_0007s10950g [Populus trichocarpa]
POPTR_010G012400v3	907	3824.43	1573.93	-1.015	3.99×10^{-6}	Down	gi∣224110416∣ref∣XP_002315514.1∣/4.4e-69/hypothetical protein POPTR_0010s01620g [Populus trichocarpa]
POPTR_001G406400v3	954	0	10.51	1.02862	2.13×10^{-5}	Up	-
POPTR_011G129100v3	938	176.5	55.19	-1.082	1.53×10^{-5}	Down	gi∣566195260∣ref∣XP_002316949.2∣/7.8e-94/hypothetical protein POPTR_0011s13280g [Populus trichocarpa]
POPTR_002G128900v3	1246	42.86	13.59	-1.2147	3.72×10^{-7}	Down	gi∣224063553∣ref∣XP_002301201.1∣/4.1e-119/hypothetical protein POPTR_0002s13030g [Populus trichocarpa]
POPTR_009G142300v3	1034	313.99	58.36	-1.7804	3.03×10^{-14}	Down	gi∣566187971∣ref∣XP_006379301.1∣/3.1e-183/hypothetical protein POPTR_0009s14430g [Populus trichocarpa]
POPTR_001G060500v3	1142	50.86	145.13	1.14677	7.36×10^{-10}	Up	gi∣566148756∣ref∣XP_002299330.2∣/2.8e-121/hypothetical protein POPTR_0001s13450g [Populus trichocarpa]//gi∣566148756∣ref∣XP_002299330.2∣/9.0e-112/hypothetical protein POPTR_0001s13450g [Populus trichocarpa]
POPTR_011G079500v3	1412	2523.75	7264.69	1.06205	2.09×10^{-6}	Up	gi∣224114357∣ref∣XP_002316737.1∣/2.4e-152/Chlorophyll a-b binding protein 2 [Populus trichocarpa]
POPTR_017G128800v3	1101	140.73	56.68	-1.0332	7.36×10^{-6}	Down	gi∣566211027∣ref∣XP_006372587.1∣/4.0e-107/hypothetical protein POPTR_0017s03000g [Populus trichocarpa]//gi∣566211029∣ref∣XP_006372588.1∣/1.3e-116/hypothetical protein POPTR_0017s03000g [Populus trichocarpa]
POPTR_001G077300v3	739	27.43	2.3	-1.0201	7.65×10^{-6}	Down	gi∣224057934∣ref∣XP_002299397.1∣/6.6e-30/hypothetical protein POPTR_0001s12020g [Populus trichocarpa]
POPTR_005G200900v3	1173	1170.06	3113.1	1.08373	4.63×10^{-11}	Up	gi∣566172683∣ref∣XP_002307539.2∣/8.4e-79/hypothetical protein POPTR_0005s22280g [Populus trichocarpa]//gi∣566172683∣ref∣XP_002307539.2∣/9.4e-81/hypothetical protein POPTR_0005s22280g [Populus trichocarpa]
POPTR_006G118300v3	1502	113.27	285.42	1.03551	6.70×10^{-11}	Up	gi∣566175817∣ref∣XP_006381340.1∣/1.7e-201/hypothetical protein POPTR_0006s11970g [Populus trichocarpa]
POPTR_019G031200v3	1635	2950.61	585.42	-1.0938	1.52×10^{-5}	Down	gi∣224144743∣ref∣XP_002325398.1∣/1.4e-224/putative apyrase family protein [Populus trichocarpa]//gi∣224144743∣ref∣XP_002325398.1∣/1.4e-246/putative apyrase family protein [Populus trichocarpa]//gi∣224144743∣ref∣XP_002325398.1∣/8.6e-249/putative apyrase family protein [Populus trichocarpa]
POPTR_012G091200v3	1267	20.52	91.05	1.24457	4.75×10^{-7}	Up	gi∣566197602∣ref∣XP_006376888.1∣/2.0e-177/hypothetical protein POPTR_0012s09300g [Populus trichocarpa]//gi∣566197602∣ref∣XP_006376888.1∣/4.6e-210/hypothetical protein POPTR_0012s09300g [Populus trichocarpa]//gi∣566197602∣ref∣XP_006376888.1∣/5.4e-135/hypothetical protein POPTR_0012s09300g [Populus trichocarpa]
POPTR_001G448700v3	1453	263.77	712.89	1.19957	3.62×10^{-26}	Up	gi∣566155014∣ref∣XP_002298964.2∣/3.4e-249/hypothetical protein POPTR_0001s45280g [Populus trichocarpa]

POPTR_006G028000v3	1660	389.14	1801.97	1.55523	5.85×10^{-13}	Up	gi|566174170|ref|XP_002307941.2|/5.5e-183/hypothetical protein POPTR_0006s02880g [Populus trichocarpa]
POPTR_010G090500v3	1714	251.96	867.13	1.3054	1.58×10^{-10}	Up	gi|224108075|ref|XP_002314711.1|/4.1e-158/hypothetical protein POPTR_0010s10090g [Populus trichocarpa]
POPTR_019G092600v3	2416	280.78	121.52	-1.039	1.27×10^{-6}	Down	gi|224145287|ref|XP_002325591.1|/0.0e+00/acyl-CoA oxidase family protein [Populus trichocarpa]
POPTR_012G084800v3	2383	399.67	974.92	1.05213	4.95×10^{-13}	Up	gi|224119412|ref|XP_002318065.1|/0.0e+00/ferric reductase-like transmembrane component family protein [Populus trichocarpa]//gi|224119412|ref|XP_002318065.1|/1.0e-254/ferric reductase-like transmembrane component family protein [Populus trichocarpa]
POPTR_003G110200v3	497	2.65	16.43	1.03429	4.85×10^{-5}	Up	gi|224074669|ref|XP_002304416.1|/3.7e-75/hypothetical protein POPTR_0003s10960g [Populus trichocarpa]
POPTR_001G407800v3	2355	1042.55	2911.57	1.06733	1.05×10^{-7}	Up	gi|566154176|ref|XP_006370343.1|/0.0e+00/hypothetical protein POPTR_0001s41830g [Populus trichocarpa]//gi|566154176|ref|XP_006370343.1|/4.0e-273/hypothetical protein POPTR_0001s41830g [Populus trichocarpa]//gi|566154176|ref|XP_006370343.1|/6.5e-204/hypothetical protein POPTR_0001s41830g [Populus trichocarpa]
POPTR_018G091400v3	1285	179.61	52.65	-1.4057	5.88×10^{-11}	Down	gi|566215085|ref|XP_006372084.1|/3.5e-131/hypothetical protein POPTR_0018s09880g [Populus trichocarpa]
POPTR_001G071000v3	1537	753.67	334.24	-1.0321	1.45×10^{-6}	Down	gi|224053607|ref|XP_002297895.1|/9.8e-179/× 10XGT1 family protein [Populus trichocarpa]
POPTR_006G141100v3	1008	17.47	66.46	1.26196	5.76×10^{-8}	Up	gi|566176377|ref|XP_006381614.1|/2.4e-73/hypothetical protein POPTR_0006s14330g [Populus trichocarpa]
POPTR_008G214800v3	760	38.01	7.39	-1.2269	1.36×10^{-6}	Down	gi|566185440|ref|XP_006380199.1|/1.9e-52/glutaredoxin family protein [Populus trichocarpa]
POPTR_015G071500v3	1102	106.54	430.23	1.30613	1.47×10^{-8}	Up	gi|224133432|ref|XP_002321566.1|/2.7e-50/hypothetical protein POPTR_0015s08290g [Populus trichocarpa]//gi|224133432|ref|XP_002321566.1|/3.0e-35/hypothetical protein POPTR_0015s08290g [Populus trichocarpa]//gi|224133432|ref|XP_002321566.1|/6.5e-59/hypothetical protein POPTR_0015s08290g [Populus trichocarpa]//gi|224133432|ref|XP_002321566.1|/7.1e-46/hypothetical protein POPTR_0015s08290g [Populus trichocarpa]//gi|224133432|ref|XP_002321566.1|/7.3e-41/hypothetical protein POPTR_0015s08290g [Populus trichocarpa]
POPTR_001G013400v3	2452	115.79	10.2	-1.7354	8.29×10^{-12}	Down	gi|743886688|ref|XP_011037917.1|/0.0e+00/PR× 10DICT× 10D: protein NRT1/ PTR FAMILY 7.1-like [Populus euphratica]
POPTR_017G080100v3	1488	76.63	210.51	1.14271	3.46×10^{-11}	Up	gi|566212775|ref|XP_002323843.2|/2.0e-210/hypothetical protein POPTR_0017s11650g [Populus trichocarpa]
POPTR_011G129700v3	2273	221.53	1202.92	1.85379	3.95×10^{-20}	Up	gi|224115146|ref|XP_002316954.1|/0.0e+00/DWARF IN LIGHT 1 family protein [Populus trichocarpa]
POPTR_014G017300v3	1063	250.33	62.32	-1.2747	3.69×10^{-7}	Down	gi|224127280|ref|XP_002320035.1|/3.4e-113/zinc-finger family protein [Populus trichocarpa]//gi|743786678|ref|XP_011028801.1|/1.7e-115/PR× 10DICT× 10D: zinc finger protein ZAT10-like [Populus euphratica]
POPTR_001G028100v3	2206	333.17	1538.98	1.84763	6.42×10^{-33}	Up	gi|224057278|ref|XP_002299202.1|/2.7e-218/hypothetical protein POPTR_0001s06550g [Populus trichocarpa]
POPTR_008G050700v3	1056	38.9	7.33	-1.0542	2.24×10^{-5}	Down	gi|224098284|ref|XP_002311146.1|/1.7e-111/Gamma-tonoplast intrinsic protein 3 [Populus trichocarpa]//gi|224098284|ref|XP_002311146.1|/2.7e-137/Gamma-tonoplast intrinsic protein 3 [Populus trichocarpa]//gi|224098284|ref|XP_002311146.1|/2.9e-129/Gamma-tonoplast intrinsic protein 3 [Populus trichocarpa]
POPTR_006G153300v3	2718	433.37	167.89	-1.2137	4.65×10^{-9}	Down	gi|566176612|ref|XP_002309233.2|/0.0e+00/hypothetical protein POPTR_0006s15820g [Populus trichocarpa]
POPTR_010G010700v3	1161	133.45	491.28	1.27033	1.43×10^{-8}	Up	gi|743924650|ref|XP_011006451.1|/1.3e-192/PR× 10DICT× 10D: bifunctional epoxide hydrolase 2-like [Populus euphratica]
POPTR_015G133700v3	1551	116.67	339.59	1.18475	1.02×10^{-11}	Up	gi|224134589|ref|XP_002321860.1|/9.1e-275/hypothetical protein POPTR_0015s14120g [Populus trichocarpa]
POPTR_017G012100v3	1832	919.41	78.72	-2.3427	1.69×10^{-21}	Down	gi|566210478|ref|XP_006372325.1|/7.2e-267/Mannose/glucose-specific lectin family protein [Populus trichocarpa]
POPTR_003G190600v3	1604	1653.92	135.87	-1.066	3.07×10^{-6}	Down	gi|566256059|ref|XP_006388026.1|/8.1e-243/hypothetical protein POPTR_0393s00210g [Populus trichocarpa]
POPTR_013G014300v3	1081	3.96	24.22	1.15984	4.99×10^{-6}	Up	gi|566198835|ref|XP_002318984.2|/8.6e-84/hypothetical protein POPTR_0013s01580g [Populus trichocarpa]
POPTR_001G154100v3	1181	45.5	16.39	-1.0123	4.06×10^{-5}	Down	gi|566149142|ref|XP_006368978.1|/3.7e-150/hypothetical protein POPTR_0001s15390g [Populus trichocarpa]
POPTR_003G036700v3	772	4.45	30.72	1.43388	7.56×10^{-9}	Up	-
POPTR_017G010900v3	1648	419.21	51.23	-1.8914	5.25×10^{-14}	Down	gi|566211322|ref|XP_006372713.1|/6.8e-68/hypothetical protein POPTR_0017s043602g, partial [Populus trichocarpa]//gi|743915676|ref|XP_011001798.1|/5.5e-258/PR× 10DICT× 10D: mannose/glucose-specific lectin-like isoform X3 [Populus euphratica]//gi|743915676|ref|XP_011001798.1|/9.7e-258/PR× 10DICT× 10D: mannose/glucose-specific lectin-like isoform X3 [Populus euphratica]
POPTR_008G139300v3	1182	97.76	628.75	2.0774	5.65×10^{-27}	Up	gi|566183858|ref|XP_002312482.2|/1.8e-192/hypothetical protein POPTR_0008s13870g [Populus trichocarpa]
POPTR_007G045100v3	2555	663.8	299.11	-1.1125	7.73×10^{-12}	Down	gi|566180673|ref|XP_002310134.2|/0.0e+00/subtilase family protein [Populus trichocarpa]
POPTR_006G144500v3	3419	944.51	322.81	-1.1933	5.63×10^{-7}	Down	gi|566176452|ref|XP_002308268.2|/0.0e+00/hypothetical protein POPTR_0006s14680g [Populus trichocarpa]
POPTR_013G150900v3	3115	19.53	91.16	1.49048	5.39×10^{-11}	Up	gi|566201368|ref|XP_006376542.1|/0.0e+00/hypothetical protein POPTR_0013s14720g [Populus trichocarpa]
POPTR_011G048200v3	1032	1.33	13.91	1.12295	9.28×10^{-6}	Up	gi|224116140|ref|XP_002317223.1|/9.2e-197/sulfotransferase family protein [Populus trichocarpa]
POPTR_006G206800v3	1027	13.31	49.44	1.17636	5.73×10^{-7}	Up	gi|224091933|ref|XP_002309405.1|/7.9e-154/alcohol dehydroge family protein [Populus trichocarpa]

POPTR_017G033600v3	1933	21.08	5.68	-1.1358	7.04×10^{-6}	Down	gi|566211730|ref|XP_006372917.1|/0.0e+00/hypothetical protein POPTR_0017s06220g [Populus trichocarpa]
POPTR_008G099800v3	1602	75.25	27.82	-1.0636	5.77×10^{-6}	Down	gi|566183210|ref|XP_002312300.2|/2.5e-174/hypothetical protein POPTR_0008s09920g [Populus trichocarpa]
POPTR_015G003100v3	1397	171.73	78.43	-1.1218	3.82×10^{-12}	Down	gi|224134961|ref|XP_002321948.1|/3.1e-116/Chain A family protein [Populus trichocarpa]//gi|224134961|ref|XP_002321948.1|/6.4e-212/Chain A family protein [Populus trichocarpa]//gi|224134961|ref|XP_002321948.1|/7.1e-147/Chain A family protein [Populus trichocarpa]
POPTR_001G472200v3	3229	37.59	169.05	1.44074	1.68×10^{-10}	Up	gi|224056903|ref|XP_002299081.1|/0.0e+00/hypothetical protein POPTR_0001s47610g [Populus trichocarpa]
POPTR_013G144200v3	1008	213.1	33.86	-1.3864	4.88×10^{-8}	Down	gi|224124580|ref|XP_002319367.1|/1.2e-26/hypothetical protein POPTR_0013s14040g [Populus trichocarpa]//gi|224124580|ref|XP_002319367.1|/3.5e-48/hypothetical protein POPTR_0013s14040g [Populus trichocarpa]
POPTR_017G012000v3	1688	116.35	15.89	-1.6159	1.91×10^{-10}	Down	gi|743915678|ref|XP_011001799.1|/4.9e-199/PR× 10DICT× 10D: 60 kDa jasmonate-induced protein-like isoform X1 [Populus euphratica]
POPTR_006G143200v3	2014	123.48	786.91	2.09621	2.85×10^{-28}	Up	gi|566176427|ref|XP_006381639.1|/3.8e-223/hypothetical protein POPTR_0006s14560g [Populus trichocarpa]
POPTR_006G177000v3	913	171.42	69.07	-1.1616	3.90×10^{-8}	Down	gi|224088376|ref|XP_002308431.1|/6.2e-94/hypothetical protein POPTR_0006s19110g [Populus trichocarpa]
POPTR_015G005900v3	1939	56.36	171.76	1.16452	3.09×10^{-8}	Up	gi|566205288|ref|XP_006374090.1|/7.0e-220/hypothetical protein POPTR_0015s00830g [Populus trichocarpa]
POPTR_001G175700v3	3407	64.84	17.37	-1.242	6.62×10^{-7}	Down	gi|566149595|ref|XP_002298123.2|/0.0e+00/ABC transporter family protein [Populus trichocarpa]
POPTR_001G453800v3	1716	107.41	394.35	1.37077	2.09×10^{-11}	Up	gi|566155104|ref|XP_002300529.2|/4.8e-114/hypothetical protein POPTR_0001s45800g [Populus trichocarpa]
POPTR_002G060800v3	1189	406.83	2819.03	2.26624	7.65×10^{-39}	Up	gi|566156501|ref|XP_006386286.1|/5.3e-94/hypothetical protein POPTR_0002s06160g [Populus trichocarpa]
POPTR_012G019500v3	2199	1.67	32.12	1.45128	1.17×10^{-8}	Up	gi|566196442|ref|XP_006376657.1|/0.0e+00/hypothetical protein POPTR_0012s02650g [Populus trichocarpa]
POPTR_006G069400v3	1023	13.01	1.33	-1.1526	2.23×10^{-6}	Down	gi|566174914|ref|XP_002308080.2|/4.5e-117/AP2 domain-containing transcription factor family protein [Populus trichocarpa]
POPTR_014G012800v3	2408	557.11	205.19	-1.2702	4.92×10^{-10}	Down	gi|743912082|ref|XP_010999912.1|/7.2e-301/PR× 10DICT× 10D: growth-regulating factor 1-like [Populus euphratica]
POPTR_010G132300v3	2735	51.98	157.92	1.03594	6.93×10^{-6}	Up	gi|566190773|ref|XP_002314898.2|/0.0e+00/× 10XS family protein [Populus trichocarpa]//gi|743797732|ref|XP_011008836.1|/0.0e+00/PR× 10DICT× 10D: phosphate transporter PHO1 homolog 1-like [Populus euphratica]
POPTR_014G140000v3	1038	87.09	34.9	-1.054	2.02×10^{-6}	Down	gi|224128702|ref|XP_002320398.1|/2.9e-108/hypothetical protein POPTR_0014s13610g [Populus trichocarpa]
POPTR_019G009200v3	1392	600.81	182.6	-1.0893	1.42×10^{-5}	Down	gi|118485710|gb|ABK94705.1|/1.4e-208/unknown [Populus trichocarpa]//gi|118485710|gb|ABK94705.1|/2.9e-165/unknown [Populus trichocarpa]
POPTR_019G118900v3	1050	90.3	39.84	-1.1536	6.01×10^{-11}	Down	gi|224145547|ref|XP_002325681.1|/1.5e-143/hypothetical protein POPTR_0019s14140g [Populus trichocarpa]
POPTR_014G126900v3	2066	4736.61	18999.4	1.51274	1.34×10^{-14}	Up	gi|224131088|ref|XP_002320998.1|/6.9e-297/endo-1 family protein [Populus trichocarpa]
POPTR_006G103800v3	434	2.62	25.77	1.12176	9.29×10^{-6}	Up	gi|566175546|ref|XP_002309195.2|/1.1e-48/hypothetical protein POPTR_0006s10470g [Populus trichocarpa]
POPTR_002G086500v3	2950	2.37	45.57	1.50878	3.06×10^{-9}	Up	gi|566156974|ref|XP_002301000.2|/0.0e+00/hypothetical protein POPTR_0002s08700g [Populus trichocarpa]
POPTR_006G207300v3	1472	11.96	87.93	1.92237	6.70×10^{-18}	Up	gi|224088438|ref|XP_002308452.1|/1.9e-225/IAA-amino acid hydrolase 1 family protein [Populus trichocarpa]//gi|224088438|ref|XP_002308452.1|/5.7e-255/IAA-amino acid hydrolase 1 family protein [Populus trichocarpa]
POPTR_010G096400v3	1866	151.92	68.39	-1.1092	4.57×10^{-10}	Down	gi|224108141|ref|XP_002314734.1|/6.7e-217/hypothetical protein POPTR_0010s10650g [Populus trichocarpa]
POPTR_T090000v3	1360	21.63	3.6	-1.2049	1.88×10^{-6}	Down	gi|566164507|ref|XP_006383950.1|/2.6e-170/hypothetical protein POPTR_0004s02310g [Populus trichocarpa]
POPTR_012G091300v3	1823	293.09	97.89	-1.2343	1.52×10^{-7}	Down	gi|224121874|ref|XP_002318694.1|/2.6e-230/pectate lyase 22 precursor family protein [Populus trichocarpa]
POPTR_001G315200v3	1608	2.58	17.12	1.06331	2.97×10^{-5}	Up	gi|743904612|ref|XP_011045686.1|/1.5e-173/PR× 10DICT× 10D: high mobility group B protein 15 [Populus euphratica]
POPTR_012G006000v3	1754	47.91	142.96	1.23675	4.38×10^{-12}	Up	gi|224121120|ref|XP_002318501.1|/2.7e-114/hypothetical protein POPTR_0012s00610g [Populus trichocarpa]
POPTR_012G138800v3	1315	53.07	19.31	-1.0601	8.94×10^{-6}	Down	gi|566198312|ref|XP_006377074.1|/2.9e-226/hypothetical protein POPTR_0012s13440g [Populus trichocarpa]
POPTR_001G160400v3	1257	68.32	22.93	-1.1833	5.98×10^{-7}	Down	gi|566149294|ref|XP_006369054.1|/4.5e-163/zinc transporter protein ZIP1 [Populus trichocarpa]//gi|743877775|ref|XP_011035492.1|/3.2e-133/PR× 10DICT× 10D: zinc transporter 1-like [Populus euphratica]//gi|743877775|ref|XP_011035492.1|/7.7e-186/PR× 10DICT× 10D: zinc transporter 1-like [Populus euphratica]
POPTR_009G111100v3	2318	364.58	987.95	1.22297	1.82×10^{-26}	Up	gi|566187463|ref|XP_006379226.1|/8.5e-265/hypothetical protein POPTR_0009s11420g [Populus trichocarpa]//gi|566187465|ref|XP_006379227.1|/0.0e+00/hypothetical protein POPTR_0009s11420g [Populus trichocarpa]//gi|566187467|ref|XP_006379228.1|/1.2e-282/hypothetical protein POPTR_0009s11420g [Populus trichocarpa]//gi|566187469|ref|XP_002313071.2|/0.0e+00/hypothetical protein POPTR_0009s11420g [Populus trichocarpa]//gi|566187471|ref|XP_006379229.1|/0.0e+00/hypothetical protein POPTR_0009s11420g [Populus trichocarpa]//gi|743794140|ref|XP_010999405.1|/0.0e+00/PR× 10DICT× 10D: uncharacterized protein LOC105107246 isoform X1 [Populus euphratica]
POPTR_002G186800v3	1795	360.28	171.95	-1.0104	4.32×10^{-8}	Down	gi|566158784|ref|XP_002302807.2|/1.8e-265/hypothetical protein POPTR_0002s18790g [Populus trichocarpa]

POPTR_016G056400v3	2489	186.84	63.19	-1.5157	3.87×10^{-21}	Down	gi|224138066|ref|XP_002322721.1|/1.7e-219/hypothetical protein POPTR_0016s05670g [Populus trichocarpa]//gi|224138066|ref|XP_002322721.1|/2.9e-252/hypothetical protein POPTR_0016s05670g [Populus trichocarpa]//gi|224138066|ref|XP_002322721.1|/3.1e-265/hypothetical protein POPTR_0016s05670g [Populus trichocarpa]//gi|224138066|ref|XP_002322721.1|/5.9e-264/hypothetical protein POPTR_0016s05670g [Populus trichocarpa]
POPTR_012G067500v3	1547	175.92	75.98	-1.1214	7.90×10^{-9}	Down	gi|224119038|ref|XP_002317970.1|/1.4e-231/hypothetical protein POPTR_0012s06920g [Populus trichocarpa]//gi|224119038|ref|XP_002317970.1|/1.7e-273/hypothetical protein POPTR_0012s06920g [Populus trichocarpa]
POPTR_006G245900v3	1838	20.98	88.93	1.05598	2.71×10^{-5}	Up	gi|566178230|ref|XP_002309582.2|/4.6e-255/hypothetical protein POPTR_0006s26210g [Populus trichocarpa]
POPTR_004G180900v3	1495	60.8	223.52	1.35953	1.20×10^{-10}	Up	gi|566167292|ref|XP_002305543.2|/4.9e-117/hypothetical protein POPTR_0004s18760g [Populus trichocarpa]//gi|566167292|ref|XP_002305543.2|/8.4e-173/hypothetical protein POPTR_0004s18760g [Populus trichocarpa]
POPTR_008G088000v3	1501	22.81	87.52	1.20105	3.70×10^{-7}	Up	gi|224101373|ref|XP_002312252.1|/1.1e-261/hypothetical protein POPTR_0008s08760g [Populus trichocarpa]
POPTR_017G050600v3	2301	32.98	97.46	1.12247	4.97×10^{-8}	Up	gi|224140765|ref|XP_002323749.1|/8.5e-206/hypothetical protein POPTR_0017s07740g [Populus trichocarpa]
POPTR_018G014600v3	1469	98.35	32.35	-1.4088	4.22×10^{-13}	Down	gi|224142011|ref|XP_002324354.1|/3.2e-211/nucleoid DNA-binding family protein [Populus trichocarpa]//gi|224142011|ref|XP_002324354.1|/4.9e-273/nucleoid DNA-binding family protein [Populus trichocarpa]
POPTR_003G030400v3	1548	83.2	30.77	-1.1043	2.96×10^{-6}	Down	gi|566160471|ref|XP_002304108.2|/1.2e-202/hypothetical protein POPTR_0003s02450g [Populus trichocarpa]
POPTR_008G065600v3	1126	967.85	419.95	-1.0554	2.08×10^{-7}	Down	gi|224101113|ref|XP_002312147.1|/2.5e-163/plasma membrane intrinsic protein 1-1 [Populus trichocarpa]//gi|224101113|ref|XP_002312147.1|/2.8e-117/plasma membrane intrinsic protein 1-1 [Populus trichocarpa]//gi|224101113|ref|XP_002312147.1|/3.7e-145/plasma membrane intrinsic protein 1-1 [Populus trichocarpa]
POPTR_011G147600v3	2423	342.58	1345.86	1.62795	2.28×10^{-23}	Up	gi|224117704|ref|XP_002317647.1|/0.0e+00/dehydration-responsive family protein [Populus trichocarpa]
POPTR_017G084800v3	938	25.88	2.24	-1.5473	8.59×10^{-10}	Down	gi|224141003|ref|XP_002323865.1|/6.8e-97/hypothetical protein POPTR_0017s12100g [Populus trichocarpa]
POPTR_005G128400v3	1860	1.66	16.23	1.19202	2.78×10^{-6}	Up	gi|743912135|ref|XP_010999939.1|/0.0e+00/PR× 10DICT× 10D: protein LYK5-like isoform X1 [Populus euphratica]
POPTR_005G045900v3	1470	9.05	39.95	1.13578	5.70×10^{-6}	Up	gi|525346296|gb|AGR50491.1|/8.8e-217/S-adenosyl-L-methionine:carboxyl methyltransferase [Populus trichocarpa]
POPTR_012G062300v3	1332	118.69	29.82	-1.1764	3.74×10^{-6}	Down	gi|118481762|gb|ABK92820.1|/2.2e-131/unknown [Populus trichocarpa]
POPTR_007G081200v3	1880	137.88	42.55	-1.0775	1.60×10^{-5}	Down	gi|224096304|ref|XP_002310601.1|/0.0e+00/hypothetical protein POPTR_0007s06570g [Populus trichocarpa]//gi|566179944|ref|XP_006380471.1|/3.0e-300/hypothetical protein POPTR_0007s06570g [Populus trichocarpa]
POPTR_002G068300v3	895	115.22	23.32	-1.0665	2.12×10^{-5}	Down	gi|224066683|ref|XP_002302179.1|/1.6e-154/hypothetical protein POPTR_0002s06890g [Populus trichocarpa]//gi|224066683|ref|XP_002302179.1|/2.3e-176/hypothetical protein POPTR_0002s06890g [Populus trichocarpa]//gi|224066683|ref|XP_002302179.1|/4.7e-112/hypothetical protein POPTR_0002s06890g [Populus trichocarpa]//gi|224066683|ref|XP_002302179.1|/6.0e-146/hypothetical protein POPTR_0002s06890g [Populus trichocarpa]//gi|224066683|ref|XP_002302179.1|/9.0e-206/hypothetical protein POPTR_0002s06890g [Populus trichocarpa]
POPTR_T131500v3	1684	1427.77	428.15	-1.1511	2.45×10^{-6}	Down	gi|566151691|ref|XP_006369711.1|/2.7e-90/Pathogenesis-related family protein [Populus trichocarpa]
POPTR_002G233000v3	908	42.18	212.46	1.14665	5.47×10^{-6}	Up	gi|566159590|ref|XP_002302893.2|/5.6e-70/hypothetical protein POPTR_0002s23410g [Populus trichocarpa]
POPTR_003G212900v3	2205	18.34	0.26	-1.3319	9.09×10^{-9}	Down	gi|224076056|ref|XP_002304892.1|/0.0e+00/hypothetical protein POPTR_0003s21800g [Populus trichocarpa]
POPTR_005G108900v3	1285	222.15	622.21	1.17396	3.01×10^{-11}	Up	gi|566170714|ref|XP_006383050.1|/2.5e-175/hypothetical protein POPTR_0005s11070g [Populus trichocarpa]
POPTR_004G015600v3	2283	36.47	10.91	-1.0105	7.06×10^{-5}	Down	gi|566164386|ref|XP_002304910.2|/0.0e+00/putative oxidosqualene cyclase family protein [Populus trichocarpa]
POPTR_012G054300v3	761	64.94	24.55	-1.0523	1.09×10^{-5}	Down	gi|566196864|ref|XP_002317903.2|/1.8e-46/protease inhibitor/seed storage/lipid transfer family protein [Populus trichocarpa]
POPTR_003G003900v3	4404	39.16	112.63	1.1389	2.74×10^{-9}	Up	gi|566259872|ref|XP_006389490.1|/0.0e+00/tRNA synthetase class I family protein [Populus trichocarpa]
POPTR_014G135300v3	3255	7.97	65.36	1.43121	1.66×10^{-8}	Up	gi|566204027|ref|XP_002320368.2|/0.0e+00/hypothetical protein POPTR_0014s12980g [Populus trichocarpa]
POPTR_011G142300v3	1383	8496.41	21375.8	1.04113	8.70×10^{-12}	Up	gi|224115376|ref|XP_002317016.1|/1.1e-68/polypeptide precursor of photosystem II family protein [Populus trichocarpa]
POPTR_006G265800v3	755	25.08	101.04	1.49921	4.72×10^{-15}	Up	gi|224092617|ref|XP_002309682.1|/2.1e-28/hypothetical protein POPTR_0006s28160g [Populus trichocarpa]
POPTR_014G149800v3	1421	20.76	1.25	-1.5369	6.53×10^{-10}	Down	gi|566204322|ref|XP_002320449.2|/1.2e-85/hypothetical protein POPTR_0014s14730g [Populus trichocarpa]
